# The tobacco-specific carcinogen-operated calcium channel promotes lung tumorigenesis via IGF2 exocytosis in lung epithelial cells

**DOI:** 10.1038/ncomms12961

**Published:** 2016-09-26

**Authors:** Hye-Jin Boo, Hye-Young Min, Hyun-Ji Jang, Hye Jeong Yun, John Kendal Smith, Quanri Jin, Hyo-Jong Lee, Diane Liu, Hee-Seok Kweon, Carmen Behrens, J. Jack Lee, Ignacio I. Wistuba, Euni Lee, Waun Ki Hong, Ho-Young Lee

**Affiliations:** 1 Creative Research Initiative Center for Concurrent Control of Emphysema and Lung Cancer, College of Pharmacy, Seoul National University, Seoul 08826, Republic of Korea; 2 Department of Molecular Medicine and Biopharmaceutical Science, Graduate School of Convergence Science and Technology, Seoul National University, Suwon, Gyeonggi 16229, Republic of Korea; 3 Departments of Thoracic/Head & Neck Medical Oncology, The University of Texas M.D. Anderson Cancer Center, Houston, Texas 77030, USA; 4 Department of Biostatics, The University of Texas M.D. Anderson Cancer Center, Houston, Texas 77030, USA; 5 Division of Electron Microscopic Research, Korea Basic Science Institute, Daejeon 34133, Republic of Korea; 6 Departments of Pathology, The University of Texas M.D. Anderson Cancer Center, Houston, Texas 77030, USA; 7 Research Institute of Pharmaceutical Sciences, College of Pharmacy, Seoul National University, Seoul 08826, Republic of Korea

## Abstract

Nicotinic acetylcholine receptors (nAChRs) binding to the tobacco-specific carcinogen 4-(methylnitrosamino)-1-(3-pyridyl)-1-butanone (NNK) induces Ca^2+^ signalling, a mechanism that is implicated in various human cancers. In this study, we investigated the role of NNK-mediated Ca^2+^ signalling in lung cancer formation. We show significant overexpression of insulin-like growth factors (IGFs) in association with IGF-1R activation in human preneoplastic lung lesions in smokers. NNK induces voltage-dependent calcium channel (VDCC)-intervened calcium influx in airway epithelial cells, resulting in a rapid IGF2 secretion via the regulated pathway and thus IGF-1R activation. Silencing nAChR, α1 subunit of L-type VDCC, or various vesicular trafficking curators, including synaptotagmins and Rabs, or blockade of nAChR/VDCC-mediated Ca^2+^ influx significantly suppresses NNK-induced IGF2 exocytosis, transformation and tumorigenesis of lung epithelial cells. Publicly available database reveals inverse correlation between use of calcium channel blockers and lung cancer diagnosis. Our data indicate that NNK disrupts the regulated pathway of IGF2 exocytosis and promotes lung tumorigenesis.

## Abstract (web summary)

The binding of tobacco-specific carcinogen 4-(methylnitrosamino)-1-(3-pyridyl)-1-butanone (NNK) to nicotinic acetylcholine receptors (nAChRs) induces calcium signalling. Here the authors show that NKK-induced calcium influx in airway epithelial cells triggers IGF2 secretion and tumourigenesis.

## Graphical abstract

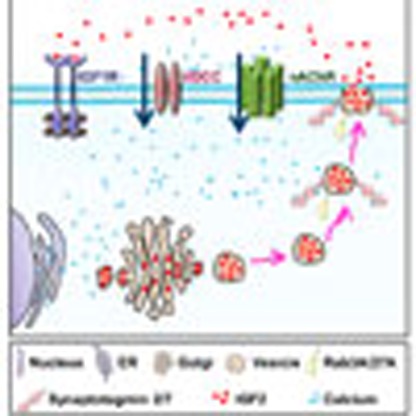

Lung cancer is the leading cause of cancer-related death worldwide^1^. Much effort has been directed towards developing effective lung cancer therapies, but without great success; thus, the need to develop novel strategies to block lung carcinogenesis is urgent. Lung cancer develops in a multistep process; it usually occurs because of years of tobacco smoking that induces genetic and epigenetic alterations in specific proto-oncogenes and tumour suppressor genes^2^. Previous studies have demonstrated that exposure to the tobacco-specific carcinogen 4-(methylnitrosamino)-1-(3-pyridyl)-1-butanone (NNK) promotes lung carcinogenesis not only through genocentric mechanisms but also through the activation of various signalling pathways that protect cells carrying damaged DNA from apoptosis^3^,^4^. We have demonstrated overexpression of insulin-like growth factors (IGFs) and deregulated activation of insulin-like growth factor 1 receptor (IGF-1R), a key mechanism for cell survival and transformation, at an early-stage of human lung carcinogenesis^5^. IGFs stimulated transformation of lung epithelial cells and development of lung cancer^5^. These findings have highlighted the role of the IGF expression and the resulting IGF-1R activation in lung cancer development; however, the mechanisms that mediate the cooperation between tissue-derived IGFs and NNK and consequently promote lung cancer development remain largely unidentified. Elucidating the biological question as to how IGFs act jointly with NNK in lung epithelial cells may provide a valuable insight that could present novel strategies for lung cancer prevention.

Previous studies have shown that NNK is a high-affinity agonist for nicotinic acetylcholine receptors (nAChRs) that are grouped into either heteromeric (comprising both α and β subunits) or homomeric (comprising only α subunits) receptors^6^. The nAChRs are widely expressed in the brain, although some studies have shown that they can be expressed in other tissues^7^. Indeed, expression of nAChRs, especially α3 and α7 nAChR, has been observed in human bronchial epithelial cells^8^,^9^,^10^,^11^. The activation of nAChRs is known to affect numerous intracellular pathways involved in cell proliferation and survival^6^,^12^,^13^. However, the mechanistic networks that mediate functional attributes of NNK-mediated nAChR activation in lung cancer are still obscure.

In this study, we present a novel mechanistic insight into the NNK-induced lung tumour formation. We identified that increase in Ca^2+^ entry resulting from binding of NNK to nAChRs and activation of voltage-dependent Ca^2+^ channels (VDCCs) leads to exocytosis of IGF2 and activation of IGF-1R, ultimately promoting lung epithelial cell transformation and lung tumour formation. These NNK-mediated events were significantly suppressed by treatment with the pharmacological and genomic antagonists that block NNK-mediated Ca^2+^ influx. Moreover, analysis of publicly available database suggests that patients prescribed calcium channel blockers (CCBs) shows significantly reduced tendency to be diagnosed as lung cancer. Together, these findings provide the novel concept of blocking Ca^2+^ channels as an approach for preventing lung cancer in smokers.

## Results

### Expression of IGFs increases during lung carcinogenesis

**Figure 1:**
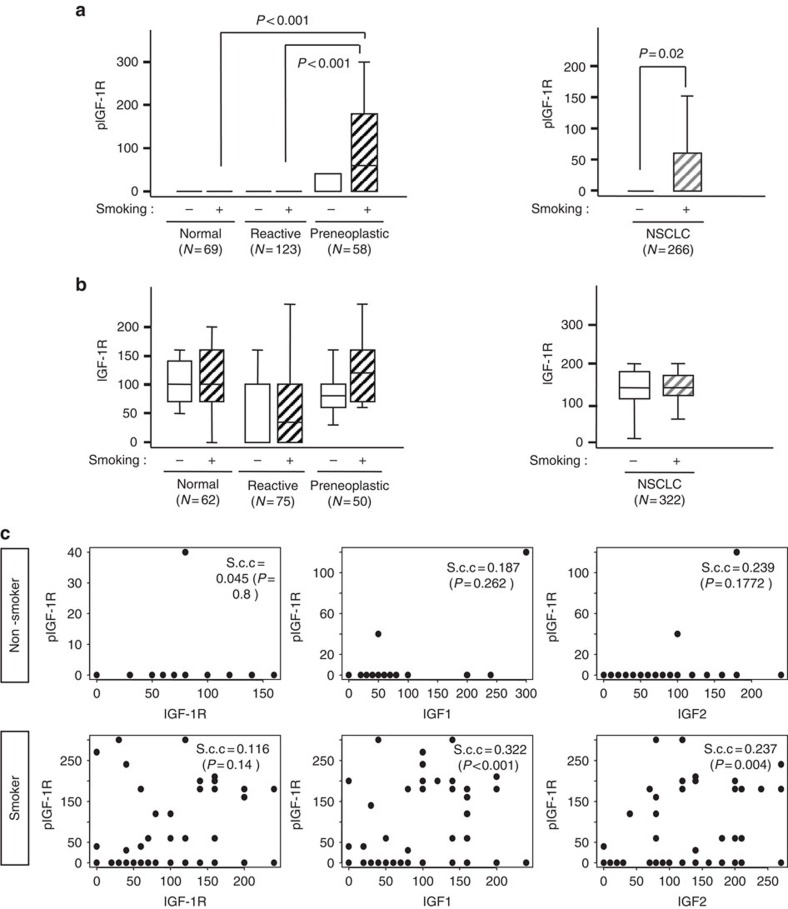
Modulation of IGF-signalling biomarkers in preneoplastic and tumour lesions in patients with NSCLC. (**a**,**b**) Comparison of IHC scoring of pIGF-1R (**a**) and IGF-1R (**b**) by histological evaluation of normal, reactive (hyperplastic and squamous metaplastic), and preneoplastic lesions (left) and non-smokers and smokers in patients with NSCLC (right). Box plots show the medians and the 25th and 75th percentiles; the bars give the ranges. Statistical significance of differences was determined with a repeated-measures model. (**c**) Linear regression analysis was performed to assess the significance of any correlation between the scoring of IGF-1R, IGF1, or IGF2 with the scoring of pIGF-1R in non-smokers (top) and smokers (bottom). The significance of the correlations was evaluated with the Spearman's rank correlation coefficient; correlations were considered significant when *P*<0.05.

We have previously demonstrated increased expression of IGF1, IGF2 and phosphorylated IGF-1R (pIGF-1R) in high-grade dysplastic lesions than in normal lung specimens^5^. In the current study, we analysed the previously studied 367 biopsy specimens of normal, reactive (hyperplasia and squamous metaplasia) and preneoplastic (low- and high-grade) lung specimens (*n*=367) (Supplementary Table 1 contains the patient demographics) for the correlation between these changes and a history of tobacco smoking. As shown in Fig. 1a (left), the preneoplastic specimens showed significantly greater levels of pIGF-1R than the normal and reactive specimens. Consistently, non-small-cell lung cancer (NSCLC) tissues from smokers showed significantly greater pIGF-1R levels compared with those from non-smokers (Fig. 1a, right). In contrast, IGF-1R expression did not show such a correlation with tobacco smoking (Fig. 1b). Then, we assessed the association between IGF expression and IGF-1R activation based on the history of tobacco smoking. Intriguingly, pIGF-1R level was significantly correlated with the levels of IGF1 and IGF2, but not IGF-1R, only in tissues from smokers (Fig. 1c). These findings suggested that tobacco smoking accelerates the activity of IGFs as autocrine ligands for IGF-1R activation in lung epithelial cells.

### NNK activates the IGF-1R pathway in lung epithelial cells

**Figure 2:**
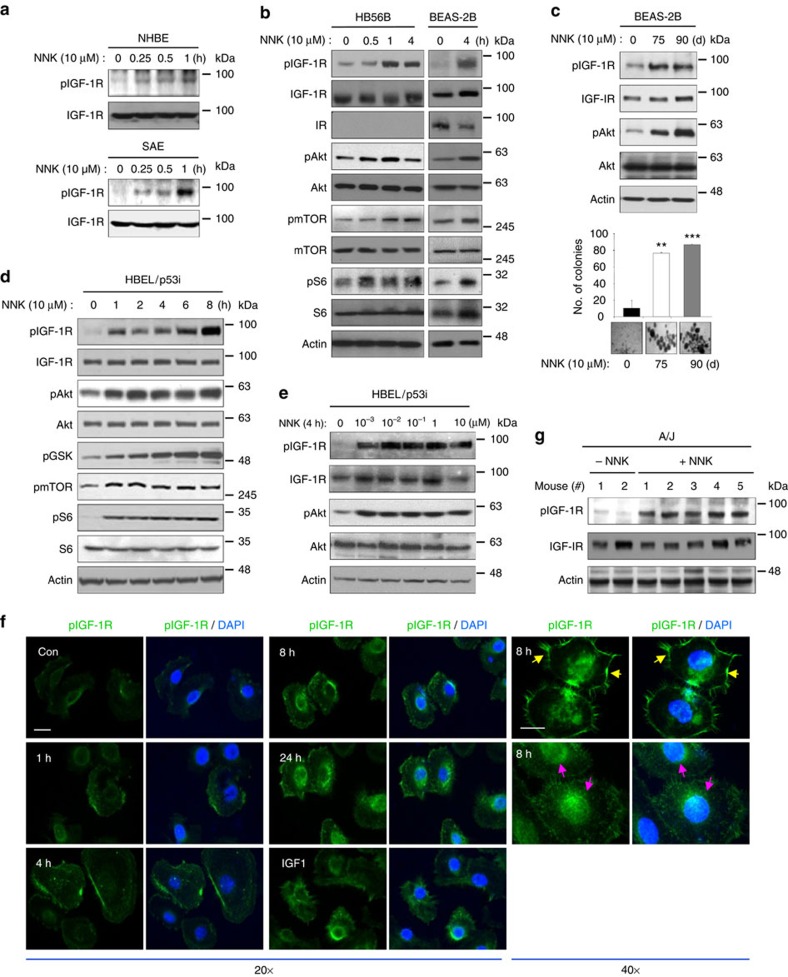
Activation of the IGF-1R signalling cascade by the tobacco carcinogen NNK in lung epithelial cells. (**a**) Normal human epithelial (top) and small airway epithelial (bottom) cells were exposed to NNK for the indicated times. Cell lysates from these cells were subjected to western blot (WB) analysis. (**b**,**d**) Time-dependent activation of IGF-1R signalling cascades in HB56B, BEAS-2B (**b**) and HBEL/p53i (**d**) cells by treatment with NNK, determined by WB analysis. (**c**) Top: BEAS-2B cells were exposed to NNK for 75 and 90 d. Activation of the IGF-1R signalling cascades were determined by WB analysis. Bottom: Anchorage-independent colony formation of BEAS-2B cells treated with NNK for 75 and 90 days (*n*=3, mean±s.d.). The statistical significance of differences was determined with the Student's *t*-test. **P<0.01; ***P<0.001, compared with the NNK-untreated group. (**e**) HBEL/p53i cells were exposed to NNK (0.001, 0.01, 0.1, 1 and 10 μM) for 4 h. NNK-activated IGF-1R signalling cascades were determined by WB analysis. (**f**) Representative fluorescence images of pIGF-1R (green), and DAPI (blue) in HBEL/p53i cells treated with NNK for the indicated times. IGF1 was used as the positive control. Yellow arrow indicates the membraneous localization and pink arrow indicates the nuclear localization. Scale bar, 10 μm. (**g**) WB analysis for IGF-1R phosphorylation in the lung from vehicle- or NNK-treated A/J mice.

To investigate the impact of IGF-1R activation in tobacco smoking-induced lung carcinogenesis, we tested the effects of NNK, a nicotine-derived, tobacco-specific nitrosamine with a high carcinogenic potential, on human lung epithelial cells. NNK induced a time-dependent IGF-1R phosphorylation in primary cultured normal human lung epithelial cells derived from large airways (NHBE), the precursor cells for squamous cell carcinomas, or small airway epithelial cells, the precursor cells for adenocarcinomas (Fig. 2a), and in various immortalized, normal human bronchial epithelial cell (HBE) cell lines, including HB56B and BEAS-2B (Fig. 2b). The NNK treatment induced propagation of the IGF-1R signalling cascades as shown by the increased phosphorylation of protein kinase B (Akt), the mammalian target of rapamycin, and ribosomal protein S6 (Fig. 2b). Chronic exposure to NNK-induced sustained IGF-1R activation along with transformed phenotypes in BEAS-2B cells as shown by increased IGF-1R and Akt phosphorylation and colony formation (Fig. 2c). Compared with these normal lung epithelial cells, premalignant HBE cell line (HBEL) carrying loss of p53 expression (HBEL/p53i) showed a prominently rapid IGF-1R and Akt phosphorylation as early as 15 min after 10 μM NNK exposure (Supplementary Fig. 1). We also confirmed NNK-induced activation of the downstream signalling cascades of the IGF-1R pathway in HBE cell lines (Fig. 2c,d). We then determined dose-dependent response of HBE cells to NNK. Activation of IGF-1R was evident in HBE cells with NNK doses as low as 10^−3^ μM, but maximal phosphorylation was observed at 10^−2^ μM (Fig. 2e; Supplementary Fig. 2). These concentrations are physiologically relevant, as average steady-state serum concentration of NNK in active smokers has been reported at 2 × 10^−4^ μM and acute increases to 10 to 100 μM in serum or to 1 mM at the mucosal surface immediately after smoking have been reported^14^. Immunofluorescence staining (Fig. 2f) revealed that pIGF-1R staining was mainly observed at the membrane at early time points (up to 4 h treatment) and then at the perinuclear region or in the nucleus at later time points (8–20 h). We additionally confirmed the NNK-induced IGF-1R activation by utilizing R^−^ (IGF-1R-null mouse embryonic fibroblast) and R^+^ (R^−^ transfected with IGF-1R) cells^15^. Western blot (Supplementary Fig. 3) and immunoprecipitation (Supplementary Fig. 4) analyses revealed that NNK increases IGF-1R phosphorylation in R^+^ cells, but not in R^−^ cells, in a time- and dose-dependent manner. NNK-induced IGF-1R phosphorylation was also confirmed *in vivo* in the lung tissues of A/J and FVB mice (Fig. 2g; Supplementary Fig. 5).

### NNK induces IGF2-dependent IGF-1R phosphorylation

**Figure 3:**
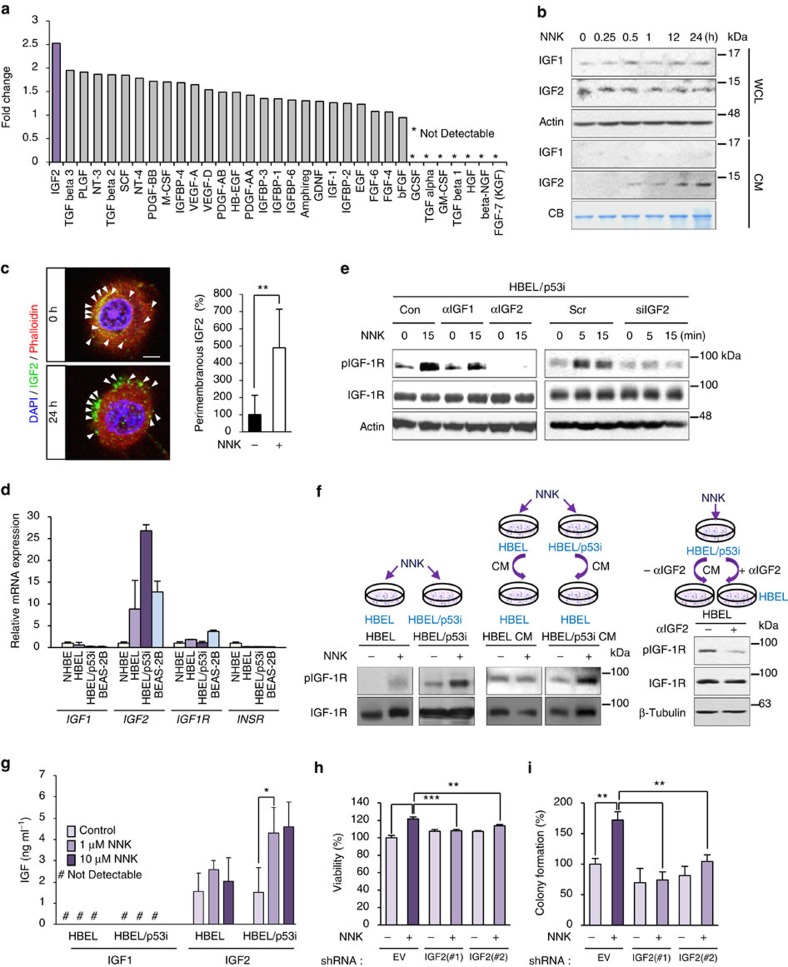
NNK-mediated IGF-1R phosphorylation is IGF2 dependent. (**a**) Secreted growth factors in the conditioned media (CM) from NNK-treated cells were determined by using human growth factor antibody array. (**b**) BEAS-2B cells were exposed to NNK for the indicated time intervals. Whole-cell lysates (WCL) or conditioned media (CM) were prepared, and NNK-induced IGF1 and IGF2 secretion was determined by Western blot (WB) analysis. Coomassie Brilliant Blue staining (CB) was used as the loading control. (**c**) Representative images of HBEL/p53i cells stained with IGF2 (green), phalloidin (red) and DAPI (blue) following 24 h culture with or without NNK (left). Graph shows the quantitative analyses of perimembranous IGF2 per field (right; mean±s.d.). Scale bar, 5 μm. (**d**) Real-time PCR analysis for determining the basal mRNA expression of IGF1, IGF2, IGF1R, and IR in NHBE, HBEL, HBEL/p53i, and BEAS-2B cells. The relative mRNA expression by comparison with the expression level in NHBE cells is depicted (*n*=3, mean±s.d.). (**e**) HBEL/p53i cells were pretreated with IGF1- or IGF2-neutralizing antibodies (left) or transfected with *IGF2* siRNAs (right). Cells were then exposed to NNK for 15 min. Cell lysates were subjected to WB analysis. (**f**) Protein lysates from HBEL or HBEL/p53i cells untreated or treated with NNK for 15 min, HBEL cells unstimulated or stimulated with CM from HBEL or HBEL/p53i cells treated with NNK for 15 min (left), and HBEL cells stimulated with CM from NNK-treated HBEL/p53i cells in the presence or absence of IGF2-neutralizing antibody for 15 min (right) were subjected to WB analysis. (**g**) The protein amounts of IGF1 and IGF2 in CM from HBEL or HBEL/p53i cells were determined by ELISA (*n*=3, mean±s.d.). (**h**,**i**) Modulation of cell viability (*n*=4, mean±s.d.) (**h**) and anchorage-dependent colony formation (*n*=3, mean±s.d.) (**i**) of BEAS-2B cells stably transfected with control or *IGF2* shRNAs and then treated NNK. The statistical significance of differences was determined with the Student's *t*-test. **P<0.01; ***P<0.001.

We investigated the mechanisms underlying NNK-induced IGF-1R phosphorylation. Because the NNK treatment induced no change in IGF-1R levels (Fig. 2), we assessed the main NNK-derived secretion of growth factors responsible for the IGF-1R phosphorylation. We performed a proteomic-based screening of conditioned media (CM) from BEAS-2B cells exposed to NNK versus control by using a human growth factor antibody array. The protein profile revealed multiple soluble factors (Fig. 3a). Among them, IGF2 production showed the most prominent increase in CM from NNK-treated cells compared with the CM from vehicle-treated control cells. We then monitored the kinetics of IGF production in NNK-exposed BEAS-2B cells. Western blot analysis revealed that the NNK-induced extracellular increase in IGF2 amount was evident within 30 min (Fig. 3b). In contrast, intracellular IGF2 levels decreased slightly during the NNK exposure. IGF1 production measured as a control under the same experimental conditions remained unchanged. When treated with NNK, BEAS-2B cells, in which IGF2 overexpression was achieved by transient transfection of green fluorescent protein (GFP)-conjugated IGF2 (designated BEAS-2B/GFP-IGF2), also showed GFP-IGF2 production along with decreases in cytosolic GFP-IGF2 levels (Supplementary Fig. 6a). Consistent with the NNK induced IGF-1R phosphorylation (Fig. 2e), 10 nM and 10 μM NNK induced similar levels of IGF2 production (Supplementary Fig. 6b). Confocal microscopy further confirmed the NNK-induced redistribution of IGF2 from vesicle-like cytoplasmic compartment to perimembranous regions (Fig. 3c).

We noted that HBEL/p53i cells, which showed rapid IGF-1R phosphorylation in response to NNK treatment (Supplementary Fig. 1), revealed greater basal mRNA expression levels of *IGF2*, but not *IGF1*, *IGF1R* and *INSR*, compared with the non-immortalized and immortalized normal lung epithelial cells (Fig. 3d). In accordance with greater *IGF2* transcription, NNK exposure induced greater levels of IGF-1R phosphorylation in HBEL/p53i cells than in HBEL cells. The NNK-induced IGF-1R phosphorylation in HBEL/p53i and BEAS-2B cells was attenuated by transfection with *IGF2* siRNA or by treatment with a neutralizing antibody against IGF2 (αIGF2) (Fig. 3e; Supplementary Fig. 7). When added to untreated HBEL cells, the conditioned media (CM) from NNK-treated HBEL/p53i cells induced a prominent IGF-1R phosphorylation compared with those from NNK-treated HBEL cells (Fig. 3f, left). Moreover, the CM from the NNK-stimulated HBEL/p53i cells induced a markedly decreased IGF-1R phosphorylation in untreated HBEL cells in the presence of αIGF2 (Fig. 3f, right). When analysed with enzyme-linked immunosorbent assay, HBEL/p53i cells revealed significantly higher levels of IGF2 secretion compared with HBEL cells when treated with NNK (Fig. 3g). In contrast, neither HBEL/p53i nor HBEL cells showed a significant change in IGF1 production after NNK exposure. We finally confirmed that the NNK-induced viability (Fig. 3h) and anchorage-dependent colony-forming ability of BEAS-2B cells were suppressed by transfection with shRNA against *IGF2* (Fig. 3i). In contrast, depleting EGF or FGF by siRNA transfection had no detectable effects on the NNK-induced IGF-1R phosphorylation (Supplementary Fig. 8a) and cell viability (Supplementary Fig. 8b) in BEAS-2B cells. These findings indicated that NNK-induced IGF-1R phosphorylation and transformation of lung epithelial cells were dependent on the secretion of IGF, particularly IGF2.

### Ca^2+^ is a key regulator of NNK-induced IGF2 secretion

**Figure 4:**
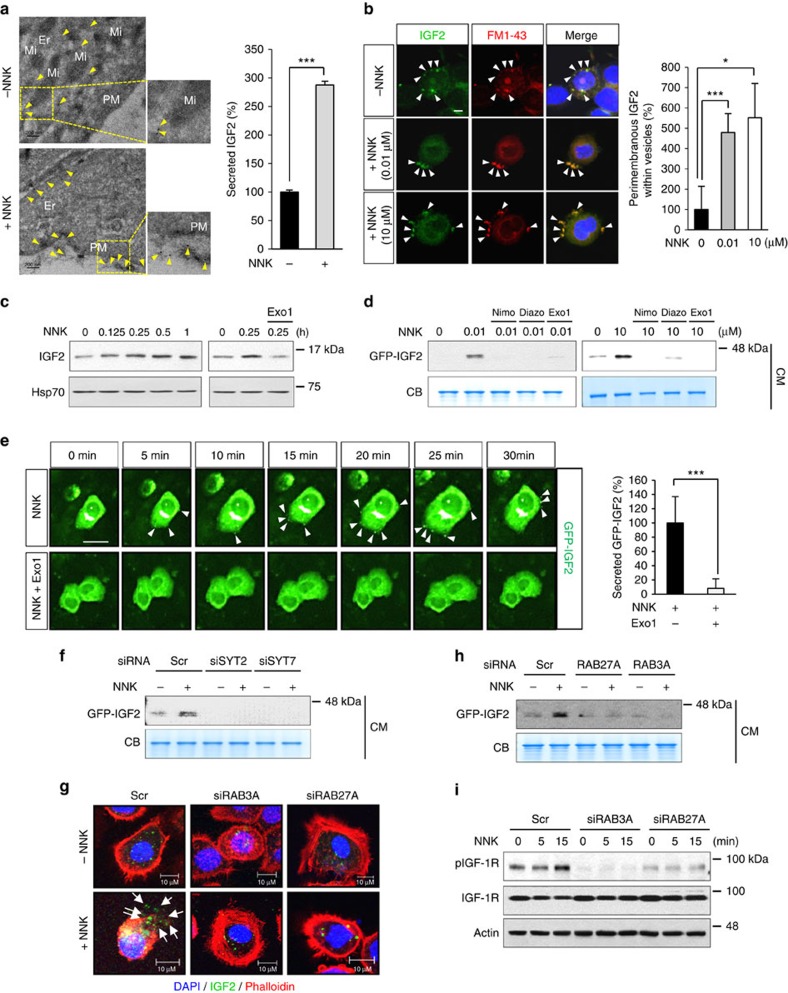
NNK-induced IGF2 secretion by regulated exocytosis. (**a**) Immuno-EM analysis of IGF2 localization in HBEL/p53i cells exposed to NNK (10 μM) for 30 min. Yellow arrow indicates the IGF2 localization (left). The secreted IGF2 was quantified and normalized to untreated with NNK as a percentage in the graph (right). Er, endoplasmic reticulum; Mi, mitochondria; PM, plasma membrane. Scale bar, 200 nm. (**b**) Confocal images of IGF2 (green), FM1-43 (red), and DAPI (blue) in HBEL/p53i cells stimulated with NNK for 15 min. White arrows indicate perimembranous IGF2 within vesicles. Graph shows the quantitative analyses of perimembranous IGF2 within vesicles per field. Scale bar, 5 μm. (**c**) BEAS-2B cells were exposed to NNK for the indicated times (left) or stimulated with NNK for 15 min with or without pretreatment with Exo1 for 3 h (right). Vesicles containing IGF2 were isolated by differential centrifugation, and IGF2 levels were determined by western blot (WB) analysis. Hsp70 was used as the loading control. (**d**) BEAS-2B/GFP-IGF2 cells were pretreated with the indicated inhibitors for 3 h, and then stimulated with NNK for 15 min. CM from these cells were subjected to WB analysis. Nimo: nimodipine; Diazo: diazoxide. (**e**) Left: BEAS-2B/GFP-IGF2 cells were pretreated with Exo1 for 3 h and then stimulated with NNK. A time-lapse imaging analysis was performed. White arrows indicate secreted GFP-IGF2. Right: secreted GFP-IGF2 out of 25 BEAS-2B cells treated with NNK or NNK and Exo1 at 30 min after NNK treatment was quantified using Harmony high content imaging and analysis software. Scale bar, 20 μm. (**f**,**h**,**i**) BEAS-2B/GFP-IGF2 cells were transfected with siRNAs targeting indicated genes, and then stimulated with NNK for 15 min. CM or WCL from these cells were subjected to WB analysis. (**g**) Confocal images of IGF2 (green), phalloidin (red) and DAPI (blue) in HBEL/p53i cells transfected with the indicated siRNAs and then treated with NNK for 15 min. White arrow indicates the secreted IGF2. Scale bar, 10 μm. Data are presented as the mean±s.d. Statistical significance was determined by Student's *t*-test. *P<0.05; ***P<0.001.

We investigated the mechanisms underlying NNK-induced IGF2 secretion. The transmission electron microscopy revealed distinct round-shaped granules without fusion with the plasma membrane in HBEL/p53i cells (Supplementary Fig. 9). Upon NNK exposure, HBEL/p53i cells showed several granules attached to the plasma membrane and secretory pods unsealed up into the apical plasma membrane or connected to the extracellular space. To assess whether the released vesicles contain IGF2, we performed immuno-electron microscopy (immuno-EM). As shown in Fig. 4a, IGF2 secretion was clearly observed in NNK-treated HBEL/p53i cells. Quantification of IGF2 secretion after visualization of the NNK-induced IGF2 exocytosis by confocal microscopy using FM 1-43 dye also displayed significant increase in IGF2 exocytosis by NNK treatment (Fig. 4b). Subcellular fractionation of NNK-treated cells by differential ultracentrifugation revealed a time-dependent increase in IGF2 protein levels in vesicles, and pretreatment with the exocytosis inhibitor Exo1 decreased IGF2 levels localized in the vesicles (Fig. 4c). Western blot (Fig. 4d) and live cell time-lapse imaging (Fig. 4e) analyses further revealed suppression of the NNK-induced GFP-IGF2 by treatment with exocytosis inhibitors, nimodipine diazoxide or Exo1 (ref. 16), in BEAS-2B/GFP-IGF2 cells.

We then determined whether IGF2 secretion occurred through the regulated pathway. We first verified endogenous expression of various vesicular trafficking curators, including target- and vesicle-localized soluble N-ethylmaleimide-sensitive factor attachment protein receptors (t- and v-SNAREs), and synaptotagmins (Syts), especially Syt2 and Syt7 in the HBE cells used in our studies (Supplementary Fig. 10). We also observed the expression of two small guanosine triphosphatases (GTPases), Ras-related protein 3A (Rab3A) and Rab27A, which play a major role in the late stages of vesicle exocytosis^17^. We then assessed the impact of loss of *SYT2, SYT7, RAB3A* and *RAB27A* by siRNA-mediated knockdown in the NNK-induced IGF2 secretion. Obvious decreases in NNK-induced GFP-IGF2 secretion were confirmed in the siSYT2- and siSYT7-transfected BEAS-2B/GFP-IGF2 cells compared with those transfected with the control siRNA (Fig. 4f). Immunofluorescence staining of NNK-treated HBEL/p53i cells revealed an obvious decrease in IGF2 fused to the cell membrane when transfected with siRNA against *RAB3A* or *RAB27A* (Fig. 4g). NNK-induced GFP-IGF2 secretion (Fig. 4h) and IGF-1R activation (Fig. 4i) were also decreased by loss of the Rab expression. Together, these findings suggested that exposure to NNK stimulated the regulatory pathway of IGF2 secretion, leading to IGF-1R activation in lung epithelial cells.

### NNK triggers Ca^2+^ influx via L-type VDCC

NNK is known to binds to β-adrenergic receptors (β-ARs)^18^ and nAChR, especially the α7nAChR subtype, we investigated whether β-AR and nAChR were involved in the NNK-mediated events. Pharmacologic blockade of β-AR or nAChR partially suppressed NNK-mediated IGF2 secretion. In contrast, blockade of both β-AR and nAChR induced complete suppression of the NNK-mediated IGF2 secretion (Supplementary Fig. 11). Therefore, both β-ARs and nAChR seemed to contribute to the NNK-induced effects on the IGF2 secretion. However, analysis of datasets available in Gene Expression Omnibus (GEO) revealed no significant difference in the expression of *ADRB1*, *ADRB2*, *CHRNA5* and *CHRFM7A (CHRNA7)* between non-smokers and smokers (Supplementary Fig. 12), suggesting that additional factors other than alterations in gene expression of β-AR and nAChR were likely to be involved in the NNK-mediated signalling events.

**Figure 5:**
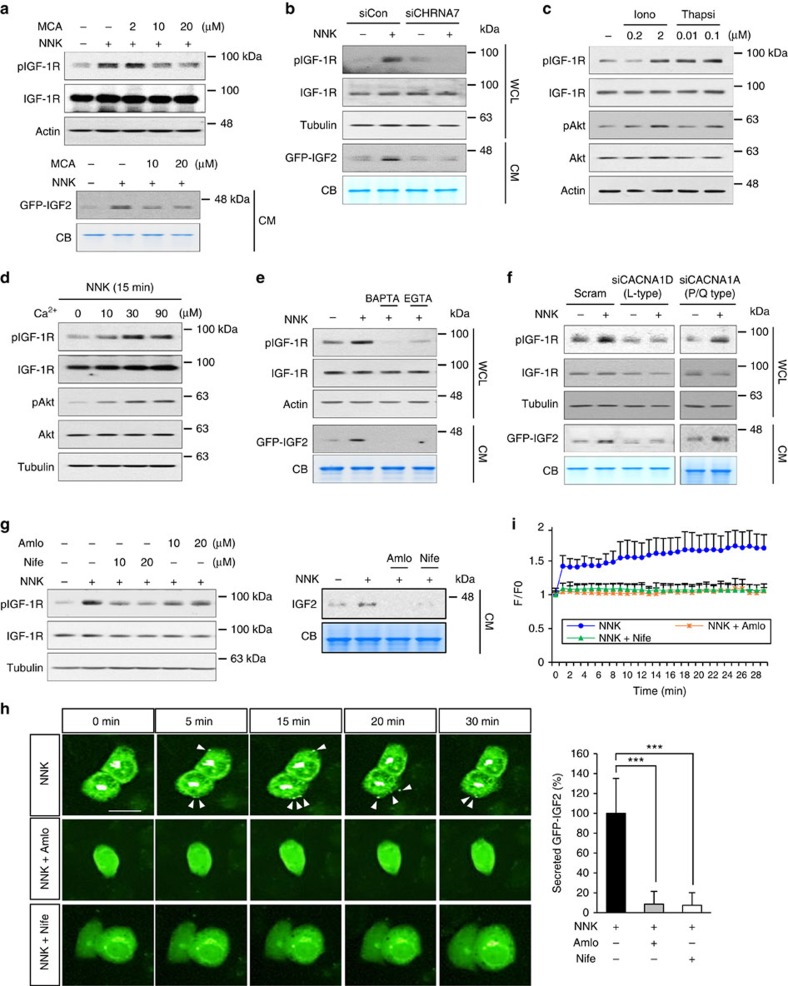
NNK-mediated increase in intracellular Ca^2+^ is important for IGF2 secretion and IGF-1R activation. (**a**) BEAS-2B/GFP-IGF2 cells were stimulated with NNK for 15 min after pretreatment with the mecamylamine (MCA) for 3 h. IGF-1R phosphorylation in WCL or IGF2 secretion in the CM were determined by western blot (WB) analysis. (**b**,**f**) BEAS-2B/GFP-IGF2 cells were transfected with indicated siRNA, and then stimulated with NNK for 15 min. IGF-1R phosphorylation in WCL or IGF2 secretion in the CM were subjected to WB analysis. (**c**,**d**) HBEL/p53i cells were treated with ionomycin (Iono) or thapsigargin (Thapsi) for 15 min (**c**) or incubated with indicated concentration of Ca^2+^ and NNK for 15 min (**d**). Activation of IGF-1R or Akt was determined by WB analysis. (**e**,**g**) HBEL/p53i or BEAS-2B/GFP-IGF2 cells were stimulated with NNK for 15 min after pretreatment with the indicated inhibitors for 3 h. IGF-1R phosphorylation in WCL or IGF2 secretion in CM were subjected to WB analysis. BAP: BAPTA-AM; Amlo: amlodipine; Nife: nifedipine. (**h**) Time-lapse imaging analysis for GFP-IGF2 secretion from BEAS-2B/GFP-IGF2 cells. Cells were pretreated with amlodipine (Amlo) or nifedipine (Nife) for 3 h and further stimulated with NNK. White arrows indicate secreted GFP-IGF2. Right: secreted GFP-IGF2 out of 25 BEAS-2B cells stimulated with NNK in the presence or absence of indicated inhibitors at 30 min after NNK treatment was quantified using Harmony high content imaging and analysis software. Data are presented as the mean±s.d. ***P<0.001, determined by Student's *t*-test. Scale bar, 20** **μm. (**i**) Time-lapse imaging of Fluo-4 AM fluorescence signals from HBEL/p53i cells. Cells were pretreated with amlodipine (Amlo; 1 μM) or nifedipine (Nife; 1 μM) for 3 h, and were exposed to NNK (10 μM) for 30 min. The changes in fluorescence intensity were plotted to show the change in intracellular Ca^2+^ levels. Data were normalized to the average fluorescence intensity measured before NNK treatment in the HBEL/p53i cells (*n*=4, mean±s.d.).

Based on the previous findings indicating the role of β-AR and nAChRs in calcium signals^19^,^20^, we postulated that NNK-stimulated Ca^2+^ influx could have been involved in the NNK-induced IGF2 secretion and IGF-1R phosphorylation. Indeed, 10 nM and 10 μM NNK increased intracellular Ca^2+^ amount in a similar level (Supplementary Fig. 13a). The NNK-induced increase in IGF-1R phosphorylation, IGF2 secretion, and intracellular Ca^2+^ level were inhibited by treatment with the AChR inhibitor mecamylamine (MCA) (Fig. 5a; Supplementary Fig. 13b) or by transfection with *CHRNA7*-specific siRNA (Fig. 5b). Consistently, boosting the intracellular Ca^2+^ levels by treatment with ionomycin (a Ca^2+^ ionophore)^21^ or thapsigargin (a non-competitive inhibitor of the sarco/endoplasmic reticulum Ca^2+^-ATPase)^22^ increased IGF-1R and Akt phosphorylation in HBEL/p53i cells (Fig. 5c). Moreover, HBE cells exposed to NNK in medium supplemented with Ca^2+^ showed a prominent IGF-1R phosphorylation compared to those exposed to NNK in Ca^2+^-free medium (Fig. 5d). Conversely, treatment with the intracellular (BAPTA-AM) and extracellular (EGTA) Ca^2+^ chelators suppressed NNK-induced increases in pIGF-1R and extracellular IGF2 levels below control levels (Fig. 5e). The complete abolition of NNK-induced IGF2 release and IGF-1R phosphorylation by extracellular EGTA (Fig. 5e) suggested that the NNK-induced Ca^2+^ signal was from influx of extracellular Ca^2+^ and not release of internal stores. However, we reasoned that NNK-induced Ca^2+^ signal could be resulting in part from the intracellular Ca^2+^ that could have been released from intracellular storage in response to extracellular Ca^2+^-induced intracellular messengers. Indeed, the NNK-induced IGF2 secretion and IGF-1R phosphorylation were partially decreased by pretreatment with 2-APB (an IP_3_ receptor antagonist) (Supplementary Fig. 14). Therefore, it is likely that release of Ca^2+^ from internal stores contributes at least in part to the NNK-induced IGF2 secretion and IGF-1R phosphorylation in lung epithelial cells. We also observed that addition of BAPTA-AM reduced IGF2 and pIGF-1R below control levels (Fig. 5e) and decreased pIGF-1R and extracellular IGF2 levels even in the absence of NNK (Supplementary Fig. 15). These findings suggest that IGF2 release and IGF-1R activation can be induced by basal intracellular Ca^2+^ levels, indicating the crucial role of Ca^2+^ signal in the regulation of IGF-1R activation mediated by IGF2 secretion.

Because the nAChRs belong to the ligand-gated ion channel superfamily and mediate Ca^2+^ influx directly through nAChRs and indirectly through voltage-dependent Ca^2+^ channels (VDCCs) upon nAChR-induced depolarization^19^, we assessed the potential implication of VDCC in NNK-induced IGF2 secretion. VDCC channels have been found in excitable cells, such as nerve and muscle cells, but are mostly excluded from nonexcitable cells^23^. Hence, we analysed the expression of VDCC components in lung epithelial cells. VDCC is composed of five subunits: α1 subunit forms central pore of VDCC; α2 subunit is connected with δ subunits as a disulfide-linked glycoprotein dimer and attached to the membrane; β subunit is an intracellular subunit and has no transmembrane segments; γ subunit is a glycoprotein and has four transmembrane segments^24^,^25^. We detected transcripts of several VDCC subunits in HBE cells (Supplementary Fig. 16). Because α1 subunit is a principal transmembrane subunit^24^, we focused on α1 subunit for further analysis. The expression profiling followed by sequencing confirmation revealed expression of L-type (Ca_v_1.3; CACNA1D) and P/Q-type (Ca_v_2.1; CACNA1A) of α1 subunit in all of the HBE cell lines used in the study. We found that siRNA-mediated depletion of *CACNA1D*, but not *CACNA1A*, completely suppressed NNK-induced IGF-1R phosphorylation and IGF2 secretion (Fig. 5f). Time-lapse imaging analyses of BEAS-2B/GFP-IGF2 cells further revealed that the *CACNA1D* depletion completely suppressed NNK-induced IGF2 secretion (Supplementary Fig. 17). Treatment with the clinically available L-type-specific calcium channel blockers (nifedipine and amlodipine) also induced an almost complete suppression of NNK-induced IGF-1R phosphorylation and IGF2 secretion (Fig. 5g,h; Supplementary Fig. 18a) at the dose that suppressed the NNK-induced increase in cytoplasmic Ca^2+^ (Fig. 5i; Supplementary Fig. 18b). These data suggested that Ca^2+^ entry via L-type VDCC is crucial for triggering IGF2 exocytosis. While the α7 isoform of the nAChR is a highly permeable Ca^2+^ channel, the Ca^2+^ entry through activated nAChR may be insufficient to trigger exocytosis. We tested the notion by using a high-[K^+^] to elicit IGF2 exocytosis. Incubation of cells with a high-[K^+^] led to increases in IGF2 secretion and IGF-1R phosphorylation, which were completely suppressed by pretreatment with amlodipine or nifedipine (Supplementary Fig. 19). These findings suggest that the key role of NNK-induced nAChR activation is to activate VDCCs by depolarization of the cell membrane, and the Ca^2+^ entry through activated nAChR may be insufficient to trigger IGF2 exocytosis. In addition, increase of intracellular [Ca^2+^] through VDCC was likely to stimulate the regulatory pathway of IGF2 secretion, leading to IGF-1R activation in lung epithelial cells. It is known that intracellular Ca^2+^ activates various downstream signalling pathways^26^. Key players for Ca^2+^-mediated signalling are a calcium binding protein calmoduin and Ca^2+^/calmodulin-dependent protein kinases (CaMKs)^27^. Calmodulin, CaMK II, as well as Src, a downstream of α7nAChR known to be activated by interaction with calmodulin or CaMK II (refs 6, 28, 29), have been implicated in the regulation of exocytosis^30^,^31^,^32^. We found that treatment with W-7 (a calmodulin antagonist) or KN-93 (a CaMK inhibitor) suppressed the NNK-induced IGF-1R phosphorylation and IGF2 secretion (Supplementary Fig. 20). Thus, in contrast to the Src-mediated negative regulation of exocytosis at the nerve terminal^33^, activation of the calmodulin-CaMK II-Src signalling seemed to mediate IGF2 exocytosis.

### Blockade of Ca^2+^ influx prevents lung tumorigenesis

**Figure 6:**
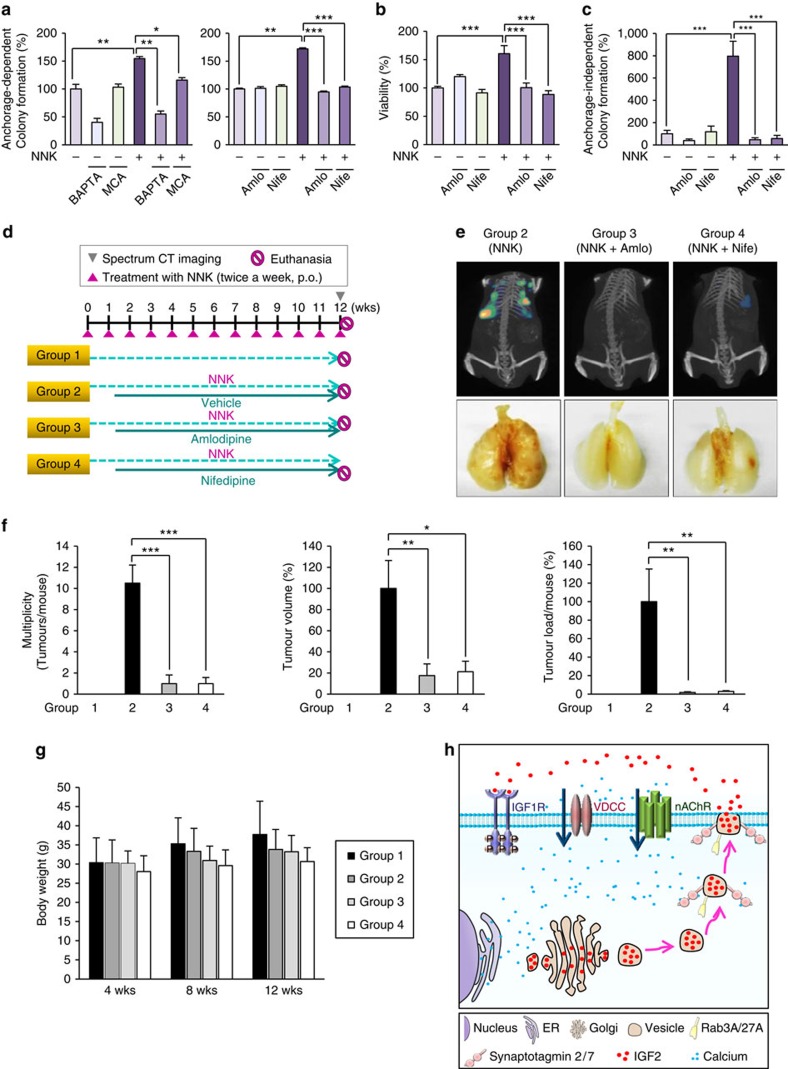
Suppression of NNK-induced acquisition of transformed phenotypes *in vitro* and lung tumour formation *in vivo* by blockade of Ca^2+^ influx. (**a**–**c**) Anchorage-dependent colony formation (*n*=3, mean±s.d.) (**a**), cell viability (*n*=3, mean±s.d.) (**b**), and anchorage-independent colony formation (*n*=4, mean±s.d.) (**c**) of BEAS-2B cells treated with NNK in combination with BAPTA-AM (BAP), mecamylamine (MCA), nifedipine (Nife), or amlodipine (Amlo). *P<0.05; **P<0.01; ***P<0.001, Student's *t*-test. (**d**) Schematic representation of NNK and CCBs (amlodipine and nifedipine) treatment schedule. Mice were treated with NNK (*n*=10) alone or in combination with amlodipine (*n*=10) or nifedipine (*n*=10) for 12 weeks. (**e**) Top: representative IVIS Spectrum CT images of mice treated with NNK (group 2), NNK and amlodipine (group 3), or NNK and nifedipine (group 4). Bottom: representative images of the lung from groups 2, 3 and 4. (**f**) Tumour multiplicity, volume, or load of groups 1, 2, 3 and 4 at 12 weeks of treatment (*n*=6, mean±s.d.). *P<0.05; **P<0.01; ***P<0.001, Student's *t*-test. (**g**) Body weight changes in groups 1 (Control), 2 (NNK), 3 (NNK+amlodipine) and 4 (NNK+nifedipine) at 4, 8 and 12 weeks of treatment. (**h**) Schematic model for the NNK-induced lung tumorigenesis *via* regulation of the IGF-1R signalling by the nAChR-VDCC-mediated calcium influx. In light of our present findings, NNK can increase intracellular Ca^2+^ level via α7nAChR and VDCC, leading to IGF2 secretion by exocytosis. Syt2, Syt7, Rab3A and Rab27A are thought to play a major role in NNK-induced exocytosis of IGF2. Finally, secreted IGF2 can activate IGF-1R signalling that promotes lung cancer formation.

We assessed the effects of inhibiting the nAChR-VDCC-mediated increase in cytosolic [Ca^2+^] on NNK-induced promotion of lung tumour formation. HBEL/p53i cells exposed to NNK showed acquisition of transformed phenotypes, as shown by significantly increased anchorage-dependent colony formation (Fig. 6a), foci formation (Supplementary Fig. 21), viability (Fig. 6b), and anchorage-independent colony formation (Fig. 6c) compared to those of vehicle-treated control cells. These NNK-induced transformed phenotypes were suppressed by treatment with a nAChR antagonist (mecamylamine), a Ca^2+^ chelator (BAPTA-AM), or CCB (nifedipine and amlodipine) (Fig. 6a–c; Supplementary Fig. 21). The capacities of CCBs to suppress NNK-induced intracellular calcium level, IGF-1R phosphorylation, and IGF2 secretion were well retained even at the low concentration within the therapeutic range (Supplementary Fig. 22)

Next, we determined the antitumour activities of CCBs in NNK-induced tumour formation in FVB mice, which have been extensively used in chemoprevention studies^34^. Figure 6d shows the treatment schedule for this experiment. FVB mice were untreated (group 1) or treated with NNK. The NNK-treated mice were subdivided into three groups, and vehicle (group 2), amlodipine (group 3) (0.8 mg kg^−1^ once a day) or nifedipine (group 4) (10 mg kg^−1^ once a day) was administered 1 week after the first dose of NNK (group 2). Twelve weeks after the first dose of NNK, we analysed representative mice from each group with *In Vivo* Imaging System (IVIS) Spectrum computed tomography (CT) to monitor lung tumour formation. The lung tumour nodule was easily detected in mice treated with NNK, but not in mice from the other groups (Fig. 6e). Gross evaluation of the lungs after all mice were killed humanely revealed no tumours in the lungs of control mice and 100% lung tumour formation in NNK-treated mice. In contrast, amlodipine- and nifedipine-treated mice had obviously decreased lung tumour nodules. Microscopic evaluation of the lungs revealed statistically significant decreases in tumour multiplicity, tumour volume, and tumour burden in the amlodipine- or nifedipine-treated mice compared to the NNK-treated mice (Fig. 6f). We observed no significant changes in body weight (Fig. 6g) and any detectable levels of organ toxicity after drug treatment. The observed antitumour activities of the drugs were associated with statistically significant decreases in IGF-1R and Akt phosphorylation in the lungs of mice exposed to NNK (Supplementary Fig. 23). Overall, these results suggested that blockade of blocking calcium channel is an effective strategy to suppress lung epithelial cell transformation and thus to prevent lung cancer development in smokers.

### CCB intake and risk of lung cancer

Up to this point, the results suggest that regulation of intracellular Ca^2+^ plays a major role in NNK-induced lung cancer development. We further analysed the Korean Health Insurance Review and Assessment Service-National Patient Sample (HIRA-NPS) database collected between 2010 and 2011 for a quantitative assessment of the cross-sectional association between CCB prescription and lung cancer diagnosis. During the 2-year study period, the estimated average number of patients from the HIRA-NPS database was 45.48 million per year (Supplementary Table 2). Of these, our study population was ∼7.53 million per year. The mean age of the study population was 62.3 years (s.d. 12.8 years) and ∼46.90% were men; 56.36% of patients were prescribed dihydropyridine (Supplementary Table 3). The most frequently prescribed CCB was amlodipine followed by nifedipine and the prevalence of lung cancer diagnosis was 0.60%. Gender, age, insurance scheme and presence of CCB prescription were significant predictors of lung cancer diagnosis. Women were less likely to be associated with lung cancer diagnosis (adjusted odds ratio (OR_adj_)=0.38; 95% confidence interval (CI): 0.35–0.41) than men (Table 1). Compared with 20–34 year old, patients who were older than 50 years were more likely to be associated with a diagnosis of lung cancer. Beneficiaries of Medicaid (OR_adj_=1.23; 95% CI: 1.07–1.40) or recipients of Veterans insurance (OR_adj_=1.93; 95% CI: 1.32–2.82) were more likely to be associated with a diagnosis of lung cancer. Patients who had a dihydropyridine CCB prescription were less likely to be associated with lung cancer diagnosis (OR_adj_=0.67; 95% CI: 0.62–0.73). These findings indicated an inverse association between CCB prescription and lung cancer diagnosis.

## Discussion

In the present study, we show a unique mechanism whereby the NNK-mediated Ca^2+^ signalling stimulates activation of the IGF-1R pathway, resulting in lung tumour development. NNK-induced IGF-1R activation appeared as a strictly Ca^2+^-dependent IGF2 release from lung epithelial cells: NNK-induced activation of nAChR results in depolarization of the cells, activation of VDCCs, and increase in the Ca^2+^ influx through VDCCs, leading to secretagogue-induced IGF2 secretion (Fig. 6h). Our study further revealed the efficacy of antagonizing Ca^2+^ channels in preventing NNK-induced transformation of lung epithelial cells *in vitro* and lung tumour formation *in vivo*. Moreover, clinically available CCB medication reduced lung cancer diagnosis in our study population. As smoking is the most prominent risk factor for lung cancer, our findings may offer new opportunities for designing interventions in clinical lung cancer chemoprevention trials.

Several prospective epidemiological studies have implied circulating IGFs in an increased risk of diverse human cancers^35^,^36^. However, prior studies have shown controversial findings for the role of circulating IGFs in lung cancer^37^,^38^,^39^. We have identified overexpression of IGFs and activation of IGF-1R in lung airway epithelial cells as early biochemical events in human lung carcinogenesis^5^,^40^. Our current data, with which we analysed the previously studied population^5^, showed a significant correlation between lung epithelial IGF expression and IGF-1R activation in a population of smokers, implying the importance of tissue-derived IGFs in lung cancer development. These findings also suggest the presence of mechanisms whereby tobacco components link IGF expression to IGF-1R activation. The tobacco-specific carcinogen NNK has been suggested to promote lung carcinogenesis not only through genocentric mechanisms but also through nAChRs-mediated activation of the Akt signalling mechanism^3^,^4^. Activation of a Src family member via NNK binding to α_3_-/α_4_- or α_7_-containing nAChRs and NNK-mediated degradation of phosphatase and tensin homologue (PTEN) have been suggested for NNK-induced Akt activation^3^,^41^,^42^. However, to date, the influence of NNK in IGF-1R activation has not yet been elucidated. Our results, including: (1) exposure to NNK-induced phosphorylation of IGF-1R and its downstream signalling mediators in various lung epithelial cells, especially those with high levels of IGF2 expression; and (2) genomic or pharmacological approaches blocking IGF2 bioavailability resulted in suppressed the NNK-induced activation of the IGF-1R pathway and acquisition of transformed phenotypes in lung epithelial cells, suggest that NNK-stimulated nAChRs couple IGF2-IGF-1R signalling to the phosphatidylinositol 3-kinase/Akt pathway. Hence, our results appear to be novel and distinct from the previously reported mechanisms underlying NNK-mediated Akt activation.

Our study provided direct evidence that NNK-induced activation of the IGF-1R pathway is mediated by regulated secretion of IGF2. Our RT-PCR analyses clearly identified lung epithelial cell expression of the various isoforms of t- and v-SNAREs that mediate the ultimate step of vesicular fusion with the outer membrane^43^, and Syts, particularly Syt2 and Syt7, which regulate membrane fusion and membrane budding reactions^44^,^45^. Syts have been shown to undertake Ca^2+^-dependent conformational changes that allow them to bind syntaxin on the cell membrane for granule fusion and membrane budding reactions^44^,^45^. Previous studies have shown that Syt2 and Syt7 are involved in insulin secretion^46^,^47^. It has been suggested that IGF2 is localized in insulin granules, and correct processing of proIGF2 into its mature form is mediated by the proconvertases that are present in insulin granules^48^. Thus, it is plausible that the same regulated pathway for insulin secretion modulates NNK-induced release of IGF2. This notion is supported by our observation that IGF2 secretion was suppressed by siRNA-mediated silencing of *SYT2* and *SYT7* or by treatment with nimodipine and diazoxide that inhibited insulin secretion^48^. Furthermore, siRNA-mediated silencing of *RAB3A* and *RAB27A*, which played a key role in the late stages of the insulin secretory pathway^49^,^50^,^51^, substantially reduced the NNK-induced exocytotic events of IGF2 and IGF-1R activation. RAB3A and RAB27A, small GTPases of the Ras superfamily, regulate the multiple steps of vesicular transport, including vesicular motility, vesicular docking to specific compartments in cells, and a membrane fusion process^50^,^51^,^52^,^53^. Therefore, a logical extension of our work is that NNK may cause insulin and IGF2 secretion. In this context, it is interesting to note the epidemiological data showing the association of cigarette smoking with insulin resistance and an increased risk for type 2 diabetes^54^. Notably, activation of nAChR leads to insulin resistance by increasing insulin receptor substrate-1 (Ser636) phosphorylation^55^. Future studies are warranted to provide the mechanistic insight to NNK-mediated insulin signalling.

We also observed the possible involvement of the β-adrenergic receptor (β-AR) in NNK-induced IGF2 secretion. The binding affinity to each receptor, shown as a EC_50_ value, were 5.8 nM for β1-AR, 128 nM for β2-AR and 30 nM for α7nAChR^18^,^56^; thus, it is likely that NNK triggers activation of both β-ARs and nAChRs, and their downstream signalling collectively operates NNK-mediated tumorigenic events. Our results clearly indicate the collaborative association of β-AR and nAChR with NNK-induced IGF2 exocytosis. Interestingly, β-AR also induces VDCC-mediated calcium influx via PKA^20^. Hence, these findings underscore that VDCC could be a common and important signalling node for NNK-induced IGF-1R activation and consequent lung tumorigenesis

The nAChR signalling pathways generally, but not invariably, stimulate Ca^2+^ influx^13^. Intracellular [Ca^2+^] has been implicated in exocytosis in addition to multiple mitogenic or apoptotic signalling pathways^57^. Our data suggest that activation of VDCCs in response to membrane depolarization caused by NNK binding to the nAChR in smokers may be responsible for the increased cytosolic [Ca^2+^] and subsequent secretagogue-induced IGF2 release and IGF-1R stimulation in lung epithelial cells. Most importantly, suppression of the Ca^2+^ influx via blockade of nAChR or VDCC effectively suppressed the NNK-stimulated IGF2-IGF-1R axis, transformation of lung epithelial cells, and lung tumour formation in mice. Finally, cross-sectional evaluation of the insurance claims data in Korea showed a significant inverse association between CCB prescription and diagnosis of lung cancer among patients with hypertension or hypertension-associated disorders. Although additional epidemiological and translational studies using longitudinal data from a large population are needed to ensure relationships between CCB medication and clinical outcomes, these findings suggest the biological plausibility of blocking Ca^2+^-mediated activation of the IGF-1R signalling cascade in lung cancer prevention.

In conclusion, we have identified a novel signalling mechanism in which the tobacco-specific carcinogen NNK stimulates transactivation of the nAChR-VDCC-IGF-1R pathway, providing premalignant lung epithelial cells with survival potential and thus inducing lung tumour formation. Given that 15% of all cancers worldwide are tobacco-related, NNK activation of the IGF-1R pathway may also help us understand the biology of these cancers. We also show the potential clinical utility of CCBs in individuals whose lung epithelial cells have overexpression of IGFs. The fact that most of the current therapies have weak efficacy in lung cancer highlights the need to identify better targets and newer strategies for preventing the disease. Currently, CCBs are considered a cornerstone therapy for the treatment of hypertension^58^. Therefore, our study has important clinical implications for the development of novel cancer chemopreventive strategies for smokers. Our data has shed new light on the emerging concept that repositioning calcium signalling-blocking drugs, such as CCBs, as an entirely novel class of IGF-1R-targeting chemopreventive agents without the potential deleterious toxicities of metabolic disorders.

## Methods

### Cell culture

Non-immortalized normal human bronchial epithelial cells (NHBEs) and small airway epithelial cells were purchased from Lifeline Cell Technology (Frederick, MD, USA). Immortalized HBE cells including HB56B^59^ and BEAS-2B^60^, were kindly provided from Dr. R Reddel (National Cancer Institute, Bethesda, MD, USA) and Dr. A. Klein-Szanto (Fox Chase Cancer Center, Philadelphia, PA, USA) , respectively. HBEL^61^ and HBEL cells carrying p53 short interfering RNA (siRNA) (HBEL/p53i)^62^,^63^ were kindly provided by Dr. John D. Minna (The University of Texas Southwestern Medical Center, Dallas, Texas, USA). Cells were cultured in K-SFM (Invitrogen, Grand Island, NY, USA) supplemented with 5 ng ml^−1^ recombinant epidermal growth factor (EGF), 50 μg ml^−1^ bovine pituitary extracts, and antibiotics. Mouse embryonic fibroblasts R^−^ and R^+^ cells were kindly provided by Dr. Renato Baserga (Columbia University, NY, USA) and were cultured in DMEM supplemented with 10% fetal bovine serum and antibiotics (all from Welgene, Daegu, Republic of Korea). Cells were maintained at 37 °C with 5% CO_2_ in a humidified atmosphere. In general, before NNK stimulation, cells were starved with supplements (for HBE cells) or serum (for R^−^ and R^+^ cells) for 1–2 days.

### Reagents

Dimethyl sulfoxide, methyllycaconitine, insulin, thapsigargin, ionomycin, crystal violet, 3-(4,5-dimethyltrizaol-2-yl)-2,5-diphenyltetrazolium bromide (MTT), 1,2-bis(2-aminophenoxy) ethane-*N,N,N*′*,N*′-tetraacetic acid tetrakis(acetoxymethyl ester) (BAPTA-AM), ethylene glycol-bis(2-aminoethylether)- *N,N,N*′*,N*′-tetraacetic acid (EGTA), 4′,6-diamino-2-phenylindole (DAPI), anti-α-tubulin antibody (Cat. No. T5168; 1:1,000 dilution) and chemicals unless otherwise indicated were purchased from Sigma-Aldrich (St. Louis, MO, USA). NNK was purchased from Toronto Research Chemicals (Toronto, Canada) or Sigma-Aldrich. Mecamylamine, Exo1, and nifedipine were purchased from Tocris Bioscience (Bristol, UK). Nimodipine, diazoxide, and amlodipine were purchased from Enzo Life Sciences (Farmingdale, NY, USA). Neutralizing antibodies against IGF1 and IGF2 were purchased from R&D Systems (Minneapolis, MN, USA). Fluo-4 AM and rhodamine-phalloidin were purchased from Invitrogen. Antibodies against phosphorylated IGF-1R (pIGF-1R) at Y1131 (Cat. No. 3021; 1:1,000 dilution) or Y1135/36 (Cat. No. 3024; 1:1,000 dilution), Akt (pAkt) at S473 (Cat. No. 4060; 1:1,000 dilution), mTOR (pmTOR) at S2448 (Cat. No. 2971; 1:1,000 dilution), S6 (pS6) at S235/236 (Cat. No. 2211; 1:1,000 dilution), ERK (pERK) at T202/Y204 (Cat. No. 9106; 1:1,000 dilution), IRS-1 (pIRS-1) at S307 (Cat. No. 1:1,000 dilution), Src (pSrc) at Y416 (Cat. No. 2109; 1:1,000 dilution), IGF-1R (Cat. No. 3027; 1:1,000 dilution), Akt (Cat. No. 9272; 1:1,000 dilution), mTOR (Cat. No. 2983; 1:1,000 dilution), S6 (Cat. No. 2217; 1:1,000 dilution), ERK (Cat. No. 9102; 1:1,000 dilution), IRS-1 (Cat. No. 2382; 1:1,000 dilution), Src (Cat. No. 2109; 1:1,000 dilution), and β-tubulin (Cat. No. 2128; 1:1,000 dilution) were purchased from Cell Signaling Technology (Danvers, MA, USA). Antibodies against IGF-1Rβ (Cat. No. sc-713; 1:1,000 dilution), IRβ (Cat. No. sc-711; 1:1,000 dilution), ERK1 (Cat. No. sc-94; 1:1,000 dilution), phospho-tyrosine (Cat. No. sc-508; 1:1,000 dilution), IGF1 (Cat. No. sc-9013; 1:1,000 dilution), IGF2 (Cat. No. sc-7435; 1:1,000 dilution), GFP (Cat. No. sc-8334; 1:1,000 dilution), Hsp70 (Cat. No. sc-1060; 1:1,000 dilution), and actin (Cat. No. sc-1615; 1:1,000 dilution) were purchased from Santa Cruz Biotechnology (Santa Cruz, CA, USA).

### Cell viability assay

BEAS-2B cells (2 × 10^3^ cells per well in 96-well plates) were treated with NNK (10 μM) in the presence or absence of inhibitors for 3 days. The MTT solution (final 500 μg ml^−1^) was added to the cells and incubated for additional 2–3 h. The cultured media were removed, and the formazan crystals were dissolved in DMSO. The absorbance was measured at 570 nm. The percentage of cell viability of each tested group was determined by comparison with vehicle-treated control.

### Anchorage-dependent colony formation assay

BEAS-2B cells (200 cells per well) were seeded into 6-well plates and then were treated with NNK (10 μM) with or without inhibitors for 2 weeks. Colonies were fixed with 100% methanol and stained with 0.002% crystal violet. Stained colonies were photographed and counted with Image J software (National Institute of Health, Bethesda, MD, USA)^64^.

### Anchorage-independent colony formation assay

BEAS-2B cells (2 × 10^3^ cells per well) were mixed with sterile low-melting agar solution (final concentration of agar: 0.35%) and immediately poured onto the 1% base agar solidified in 24-well plates. After solidification of the agar, NNK (10 μM) with or without inhibitors diluted in 0.5 ml of complete medium were added to the cells. Cells were incubated for 3 weeks, and colonies were stained with the MTT solution (final 500 μg ml^−1^), photographed, and counted with Image J software.

### Foci formation assay

HBEL/p53i cells at 70–80% confluence in 6-well plates were treated with NNK (10 μM) in the presence or absence of inhibitors for 2 weeks. The drug-containing media was replaced every 3 days. After incubation, foci were photographed and counted.

### Western Blot analysis and immunoprecipitation

Cell lysates were prepared using modified RIPA lysis buffer (50 mM Tris-HCl (pH 7.4), 150 mM NaCl, 0.25% sodium deoxycholate, 1% Triton X-100, 1 mM EDTA, 100 mM NaF, 5 mM Na_3_VO_4_, 1 mM PMSF, 1 μg ml^−1^ aprotinin, 1 μg ml^−1^ leupeptin, and 1 μg ml^−1^ pepstatin), and equivalent amounts of total cell lysate were separated by SDS–PAGE and transferred onto a PVDF membrane. Membranes were blocked with blocking buffer (3% BSA in Tris-buffered saline (TBS) containing 0.1% Tween-20 (TBST)) for 1 h at room temperature, and then were incubated with primary antibodies diluted in blocking buffer overnight at 4 °C. After washing three times with TBST, membranes were further incubated with appropriate secondary antibodies (Santa Cruz) diluted in 3% non-fat dry milk in TBST (1:5,000 dilution) for 1 h at room temperature. Membranes were washed several times with TBST and were visualized using an enhanced chemiluminescence (ECL) detection kit (Thermo Fisher Scientific, Grand Island, NY, USA or Abclon, Seoul, Republic of Korea). For analysis of the tyrosine phosphorylation status of IGF-1R or IR by immunoprecipitation, total cell or tissue lysate was incubated with specific anti-phospho-tyrosine (Santa Cruz) antibodies overnight. Protein G-Sepharose (Millipore, Temecula, CA) was then added, incubated for 4 h at 4 °C, washed five times with lysis buffer, boiled in SDS sample buffer, and the subjected to SDS–PAGE. To determine the level of IGF2 secretion in the CM, CM was prepared by concentrating the media using Amicon-Ultra (Millipore Corp., Bedford, MA, USA) and then separated by Tricine-SDS–PAGE. The uncropped scan images of important blots are shown in Supplementary Fig. 24.

### Vesicle isolation

Vesicle isolation performed as previously described^65^. In brief, BEAS-2B cells were trypsinized and resuspended in PBS. Cells were washed three times with PBS. Cells were then sonicated in ice, and vesicles were isolated by differential ultracentrifugation using TLA100.3 rotor (Beckman Coulter, Indianapolis, IN, USA).

### Reverse transcription PCR

Total RNA from cells was isolated by a phenol-chloroform extraction using Isol-RNA Lysis Reagent (5 Prime, Hilden, Germany). cDNA was synthesized from 1 μg of total RNA as a template using PrimeScript II 1st strand cDNA synthesis kit (Takara Bio Inc, Japan) and further analysed by RT-PCR or a SYBR Green-based real-time PCR with at least three replicates per group (LightCycler 480 real-time PCR system, Roche Applied Science, Penzberg, Germany). The cycling parameters for RT-PCR were as follows: initial denaturation at 94 °C for 5 min; 28–35 cycles of 95 °C for 20–30 s, 55–68 °C for 20–30 s, and 72 °C for 30–45 s; final elongation at 72 °C for 5–7 min. The thermocycler conditions for real-time PCR were as follows: pre-incubation at 95 °C for 5 min, 50–70 cycles of 95 °C for 10 s, 60 °C for 10 s, and 72 °C for 10 s, and melting curve analysis for determining reaction specificity. PCR products were separated by 2% agarose gel electrophoresis and visualized using a Gel Doc EZ System (Bio-Rad Laboratories, Hercules, CA, USA). When necessary, sequencing of PCR products was performed to confirm the specific amplification of target gene. Relative quantification of mRNA expression was performed by the comparative CT (cycle threshold) method^66^. The primer sequences used for PCR analysis are described in Supplementary Table 4. The primer sequences used for real-time PCR analysis are described in Supplementary Table 5.

### Transfection

Transient transfection was performed using Lipofectamine 2000 (Invitrogen) according to the manufacturer's instruction. For transient knockdown of Syt2, Syt7, Rab3A, Rab27A, or α7 nAChR, cells were transfected with scrambled or gene-specific targeting small interfering RNAs (siRNAs; purchased from, Shanghai GenePharma (Shanghai, China; for *SYT2, SYT7, RAB3A*, and *RAB27A* siRNAs) or Bioneer (Daejeon, Republic of Korea; for *CHRNA7* siRNA) using Lipofectamine RNAiMAX (Invitrogen) according to the manufacturer's instructions.

### ELISA

ELISA for quantification of IGF1 or IGF2 levels in the CM was performed using commercially available ELISA kits (IGF1 from R&D systems (catalogue # DG100); IGF2 from Beckman Coulter (catalogue # DSL-10-2600)) according to the manufacturer's instruction.

### Human growth factor antibody array

Analysis of secreted factors in conditioned media was performed by using human growth factor antibody array kit (RayBiotech, Norcross, GA, USA) according to the manufacturer's instruction.

### Immunohistochemistry

Lung tissues were fixed in 4% paraformaldehyde; paraffin-embedded tissue sections (4 μm) were deparaffinized and dehydrated, and then treated with 3% hydrogen peroxide to block endogenous peroxidase. Slides were incubated with the primary antibody (pIGF-1R (Cell Signaling; Cat. No. 3024; 1:200 dilution); pAkt (Cell Signaling; Cat. No. 4060; 1:200 dilution)) overnight at 4 °C. Slides were then washed with phosphate buffered saline (PBS), incubated with biotinylated secondary antibody (Dako, Carpinteria, CA, USA; Cat No. E0432; 1:500 dilution), and developed with an avidin/biotin complex kit (Vector Laboratories, Burlingame, CA, USA) and DAB detection reagents (Enzo Life Sciences). The slides were counterstained with hematoxylin.

### Immunofluorescence staining

Cells were seeded on glass coverslips and incubated in completed medium for 24 h. After overnight serum starvation, cells were treated with NNK. Cells were washed three times with PBS, fixed in 4% paraformaldehyde for 30 min at room temperature, permeabilized with 0.1% Triton X-100 for 15 min, and blocked in blocking solution (Dako) at 4 °C for 1 h. Cells were incubated with primary antibody (IGF2 (Santa Cruz; Cat. No. sc-7435; 1:100 dilution); pIGF-1R (Cell Signaling; Cat. No. 3021; 1:200 dilution)) overnight at 4 °C, washed three times with PBS, and incubated for 1 h with Alexa 488-conjugated secondary antibodies (Invitrogen; Cat. Nos. A11078 or A21203; 1:1,000 dilution) in the absence or presence of Alexa Fluor 594-phalloidin (Invitrogen; Cat. No. A12381; 1:500 dilution). After washing four times with PBS, cells were mounted with mounting solution containing the nuclear dye DAPI. Images were acquired with a confocal microscope (LSM 700; Carl Zeiss Microscopy, Jena, Germany).

### Measurement of intracellular calcium levels

HBEL/p53i were plated on glass coverslips. Cells were loaded with the Ca^2+^ indicator fluorescent dye Fluo-4 AM for 30 min at 37 °C. Cells were then loaded with NKK treatment. After treatment, cells were washed three times with PBS. Cells were fixed in 4% paraformaldehyde for 30 min at room temperature, and washed three times with PBS. Slides were mounted with mounting media containing the nuclear dye DAPI. The fluorescence images were captured with a Nikon Eclipse 80i microscope (Nikon Corp., Tokyo, Japan) equipped with a Nuance multispectral imaging system (PerkinElmer, Alameda, CA, USA).

For a time-lapse analysis of intracellular calcium levels, HBEL/p53i cells were seeded into 96-well cell carrier microplates (PerkinElmer). To follow the temporal course, intracellular calcium intensities of the fluorescent images were measured at 488 nm every 1 min for 30 min. Intracellular calcium level was detected with the Operetta high content screening system (PerkinElmer). Intracellular calcium levels are shown as F/F0 ratios, where F0 is the initial fluorescence intensity. Data analysis was performed with Harmony high content imaging and analysis software (PerkinElmer).

### Transmission electron microscopy

Cells were fixed by immersion in Karnovsky fixative for 3 h at room temperature. Fixed cells were washed with 0.05 M sodium cacodylate buffer and post-fixed in 2% osmium tetroxide and 0.1 M cacodylate buffer for 2 h at 4 °C. The cells were then dehydrated in increasing concentrations of ethanol, infiltrated with propylene oxide and embedded in Spurr's resin. Sections were cut on an ultramicrotome and examined with a JEOL 1010 TEM set to 80 kV (JEOL, Akishima, Japan).

### Immuno-electron microscopy

Cryoimmuno-EM labelling was performed as previously described^67^,^68^. Briefly, cells were fixed either in 4% paraformaldehyde and 0.05% glutaraldehyde in 0.1 M phosphate buffer (pH 7.4). The fixed cells were embedded in 10% gelatin and small blocks were infused with 2.3 M sucrose overnight and then frozen in liquid nitrogen. Ultrathin cryosections (50 nm) were cut at −120 °C with a cryo-ultramicrotome (UCT7, Leica, Vienna, Austria). The goat polyclonal antibody recognizing IGF2 (sc-7435, diluted 1:10) and protein G gold (Aurion, the Netherlands) that detect primary polyclonal antibody were used. All antibodies and gold conjugates were diluted in 0.1% BSA-c (Aurion, the Netherlands) in PBS.

### Time-lapse live cell imaging

Time-lapse fluorescence imaging was performed with the Operetta high content screening system (PerkinElmer).

### Analysis of tissue specimens

Immunohistochemical analysis of tissue microarray comprising lung biopsy specimens was performed as described previously^5^,^69^. In brief, IHC staining was performed as described above using primary antibodies against IGF1 (Santa Cruz; Cat. No. sc-9013), IGF2 (Millipore; Cat. No. 05-166), IGF-1R (Cell Signaling; Cat. No. 3027), pIGF-1R (Cell Signaling; Cat. No. 3024). The expression level of IGF1, IGF2, IGF-1R, and pIGF-1R was blindly analysed and quantified using a four-value intensity score (0, 1+, 2+ and 3+) and a percentage (0–100%) of the extent of reactivity. A final expression score was obtained by multiplying the intensity value by the extent of reactivity (range, 0–300).

### Animal experiment

All animal experiments were performed using protocols approved by the Seoul National University Institutional Animal Care and Use Committee. The mice were given food and water *ad libitum* and housed at 22±2 °C on a 12:12-h light: dark cycle in a conventionally maintained facility. 2-months-old FVB mice were randomly grouped (*n*=10 per group) and exposed to NNK (3 μmol) twice per week. After 1–2 weeks, drugs (0.8 or 10 mg kg^−1^, dissolved in water) were treated with or without NNK by oral gavage daily, 6 days a week, for additional 12 weeks. Tumour formation was monitored using the IVIS Spectrum microCT and Living Image (ver. 4.2) software (PerkinElmer). An MMPSense 680 probe (PerkinElmer; 2 nmol per 150 μl in PBS) was used to facilitate tumour monitoring. The mice were euthanized and tumour formation was evaluated and compared with that of the vehicle-treated control group. After gross evaluation of tumour formation, microscopic evaluation of formalin-fixed and paraffin-embedded lung tissue stained with hematoxylin and eosin (H&E) was also performed to measure mean tumour number (*N*) and volume (*V*) in a blinded fashion^5^. The tumour volume was calculated using the following formula: *V* (mm^3^)=(long diameter × short diameter^2^)/2, and the tumour burden was calculated using the following formula: mean tumour number (*N*) × mean tumour volume (*V*). The number and size of tumours were calculated in five sections uniformly distributed throughout each lung.

### Study population and data extraction

This study was approved by the Institutional Review Board of Seoul National University. About 97.2% of the Korean population is enrolled in the national health insurance programme coordinated by the Korean National Health Insurance Services (http://www.nhis.or.kr/). The study used publicly available national level data known as HIRA-NPS from 2010 to 2011 (serial number: HIRA-NPS-2010-0084, HIRA-NPS-2011-0110). The HIRA-NPS is collected by the Health Insurance Review and Assessment Service (HIRA) and includes about 1.3 million sample population per year. The data represents 3% of entire national population (about 46 million patients) that were selected by stratified random sampling scheme according to sex and age (Supplementary Table 2). The data provides de-identified information regarding patient- and visit- characteristics such as age, gender, diagnosis, date or services from hospital visits (both inpatient- and outpatient-visits) and prescription medications. Study population includes all patients who were aged 20 years and older and had one or more records with hypertension and hypertension related diagnoses.

### Identification of diseases and medications

The 5th and 6th revision of Korean Standard Classification of Diseases (KCD) codes, derived from the International Statistical Classification of Diseases and Related Health Problems 10th Revision, were used to identify patients with clinical conditions. Hypertension or hypertensive disorderswere operationally defined as presence of a KCD code with essential hypertension (I10), hypertensive heart disease (I11), hypertensive renal disease (I12), hypertensive heart and renal disease (I13), secondary hypertension (I15), pre-existing hypertension complicating pregnancy, childbirth and the puerperium (O10), pre-eclampsia superimposed on chronic hypertension (O11), gestational hypertension (O13), pre-eclampsia (O14), or unspecified maternal hypertension (O16). Lung cancer diagnosis included KCD codes of malignant neoplasm of bronchus and lung (C34) or carcinoma in situ of bronchus and lung (D02.2). Calcium channel blockers (CCB) included any prescription records with single or combination products composed of dihydropyridines (amlodipine, nifedipine, barnidipine, benidipine, cilnidipine, efonipine, felodipine, isradipine, lacidipine, lercanidipine, manidipine, nicardipine, nilvadipine, nimodipine, nisoldipine and nitrendipine) and non-dihydropoyridines (diltiazem and verapamil).

### Analysis of the GEO datasets

Analysis of the GEO datasets to determine alterations in gene expression in normal lung tissues was performed by using GEO dataset analysis tools. Both current and former smokers were categorized into the ‘Smokers' group.

### Statistical analysis

The statistical significance was analysed using two-sided Student's *t*-test using Microsoft Excel 2013 (Microsoft Corp., Redmond, WA, USA). *P* values <0.05 were considered statistically significant.

For analysis of the HIRA-NPS database, two-sided Student's *t*-test and analysis of variance were performed with SPSS software (SPSS, Chicago, IL). Differences between means with *P*<0.05 were accepted as statistically significant; differences between means with 0.05<*P*<0.10 were accepted as representing tendencies. We analysed the association between lung cancer risk and prescription medication of CCB therapy using meta-analysis of OR estimates. National level estimates were calculated from the sample records using the assigned weight. Variances around the estimates were computed using variables related to stratum and weighting procedures from the 2-year combined data by taking into account the complex survey design. The weighted survey estimates (that is, frequencies or proportions) with 95% confidence intervals (CI) were calculated. For assessing predictive factors, the dependent variable (lung cancer diagnosis yes/no) and independent variables related to patients (that is, age, gender, insurance scheme, and CCB use) were used to calculate adjusted odds ratios with 95% CI from a logistic regression model controlling for covariates using SAS 9.1 (SAS Institute, Cary, North Carolina).

### Data availability

The authors declare that the data supporting the findings of this study are available within the article and its Supplementary Information files.

## Additional information

**How to cite this article:** Boo, H.-J. *et al.* The tobacco-specific carcinogen-operated calcium channel promotes lung tumorigenesis via IGF2 exocytosis in lung epithelial cells. *Nat. Commun.* **7,** 12961 doi: 10.1038/ncomms12961 (2016).

## Supplementary Material

Supplementary Information Supplementary Figures 1-24, Supplementary Tables 1-5 and Supplementary References

## Figures and Tables

**Table 1:**
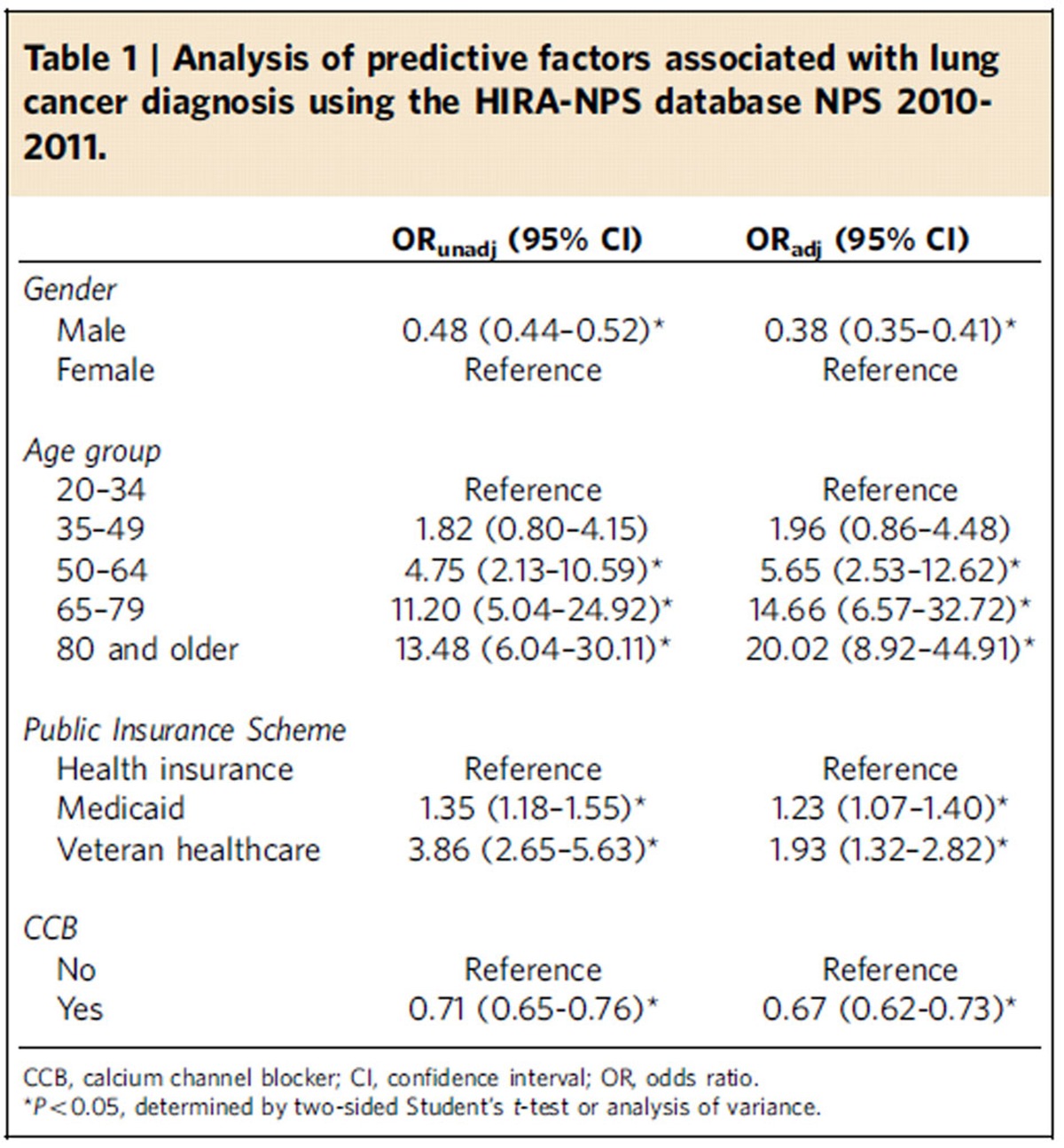
Analysis of predictive factors associated with lung cancer diagnosis using the HIRA-NPS database NPS 2010-2011.

*Reaction conditions:**1**/LiHMDS/**2**/[Pd(*η*^3^-C_3_H_5_)Cl]_2_/S-IPr·HCl=200/200/100/2.5/5; 0.1 M of ketone **1**; T=30^o^C; B/L and *dr* was determined by ^1^H NMR, *dr* is the ratio of (±)-(*syn,anti*)-**3**/other diastereoisomers; Isolated yield. †T=50 ^o^C. ‡Solvent=THF. §OBoc of **2** was replaced with OP(OEt)_2_. ||The yield was determined by ^1^H NMR.
