# Supplementary material for: The tobacco-specific carcinogen-operated calcium channel promotes lung tumorigenesis via IGF2 exocytosis in lung epithelial cells
Source: Nat Commun. 2016 Sep 26;7:12961. doi: 10.1038/ncomms12961 (PMC5052689; doi:10.1038/ncomms12961)
Supplement: Supplementary Information — Supplementary Figures 1-24, Supplementary Tables 1-5 and Supplementary References [file ncomms12961-s1.pdf]

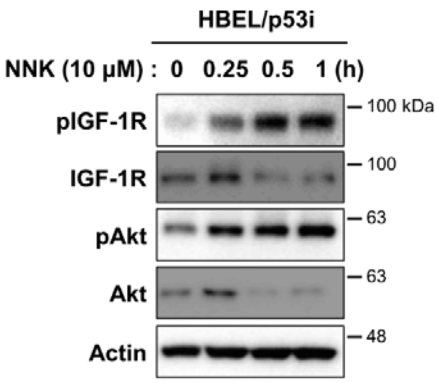

Supplementary Fig. 1. A rapid activation of IGF-1R and Akt in HBEL/p53i cells by NNK treatment. Growth factor-starved HBEL/p53i cells were exposed to NNK for the indicated times. The expression levels of total and phosphorylated IGF-1R and Akt were determined by Western blot analysis.

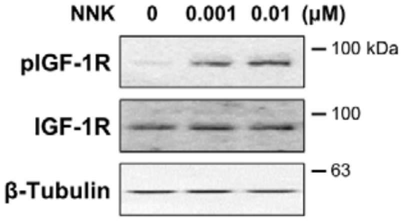

Supplementary Fig. 2. NNK-induced phosphorylation of IGF-1R in BEAS-2B cells. BEAS-2B cells were treated with 0.001 or 0.01 μM NNK for 15 min. Cell lysates were subjected to Western blot analysis.

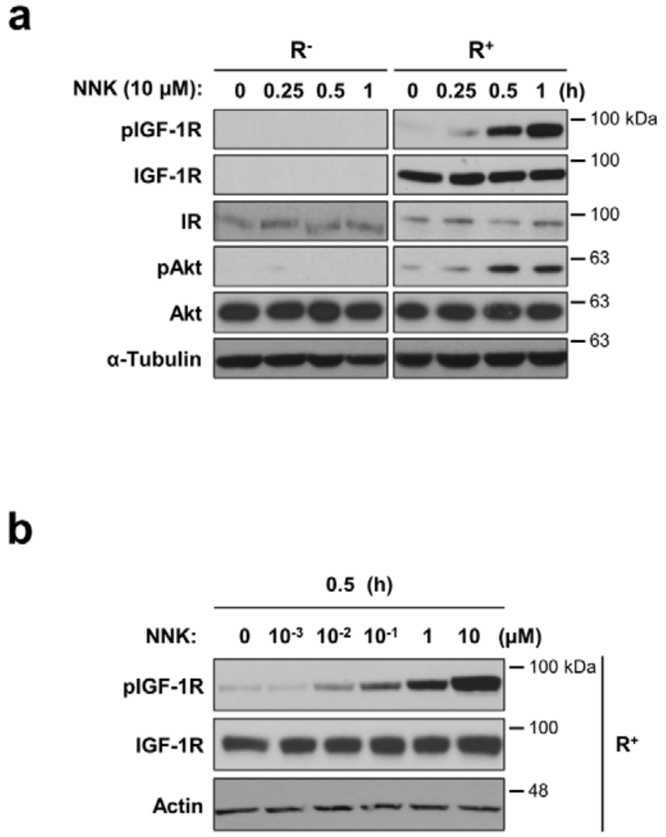

Supplementary Fig. 3. Time- and dose-dependent IGF-1R activation by NNK treatment in R$^+$ cells. Cells were exposed to NNK (10 μM) for different time intervals (**a**) or treated with various concentrations (0.001, 0.01, 0.1, 1, and 10 μM) of NNK for 0.5 h (**b**). NNK-mediated IGF-1R activation was determined by Western blot analysis.

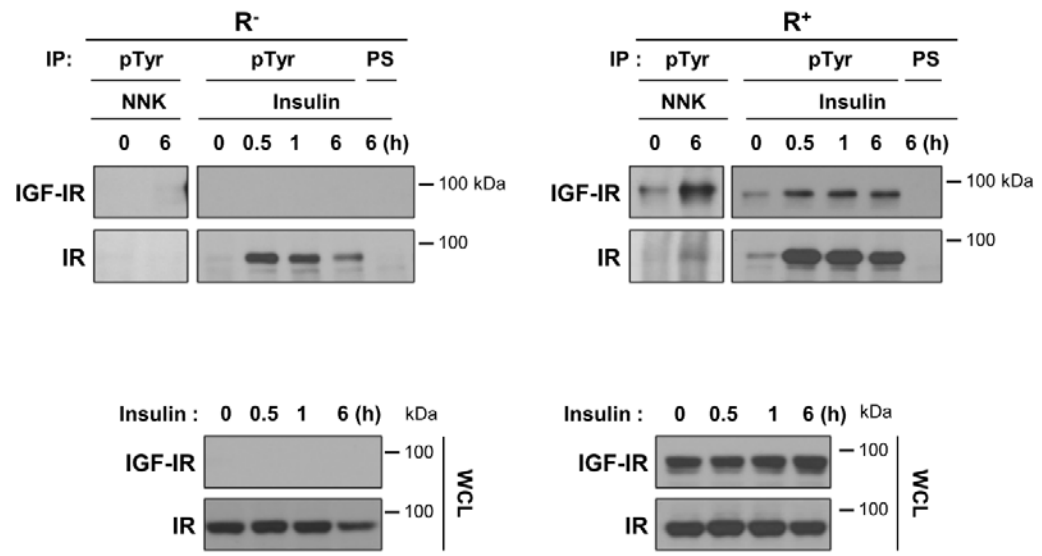

Supplementary Fig. 4. NNK specifically phosphorylates IGF-1R in R^+ cells. R^- and R^+ cells were treated with NNK or insulin for the indicated times. Cell lysates were immunoprecipitated with anti-phosphotyrosine (pTyr) antibodies and then were subjected to Western blot analysis to detect activation of IGF-1R and IR using anti-IGF-1R or anti-IR antibodies. PS: preimmune serum.

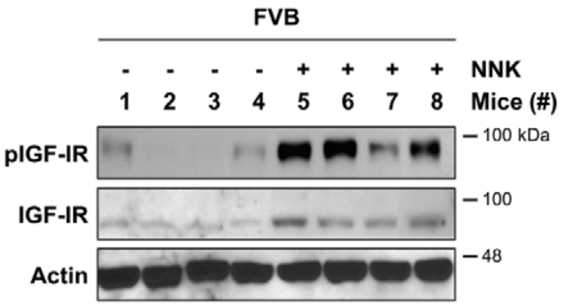

Supplementary Fig. 5. Activation of IGF-1R in the lung tissues from NNK-treated FVB mice. Lysates were prepared and analyzed by Western blot to determine the protein level of pIGF-1R in the lung from vehicle- or NNK-treated FVB mice.

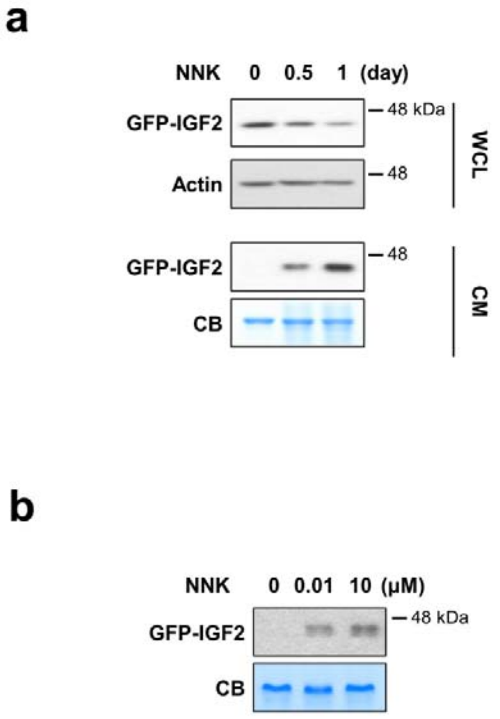

Supplementary Fig. 6. NNK-mediated increase in IGF2 secretion. BEAS-2B/GFP-IGF2 cells were exposed to 0.01 μM (b) or 10 μM (a, b) NNK for the indicated time intervals (a) or for 15 min (b). Whole-cell lysates (WCL) or conditioned media (CM) were prepared, and NNK-induced IGF2 secretion was determined by Western blot analysis. Coomassie Brilliant Blue staining (CB) was used as the loading control.

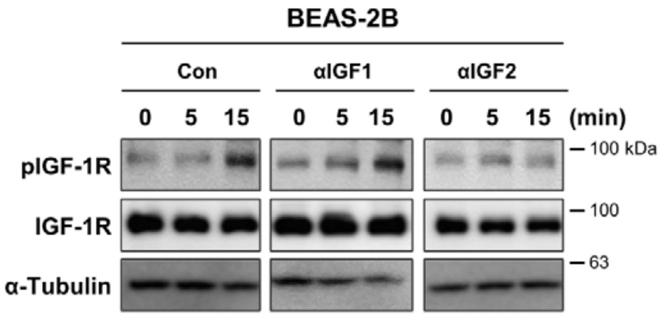

Supplementary Fig. 7. Blockade of NNK-induced IGF-1R phosphorylation by treatment with IGF2 neutralizing antibodies. BEAS-2B cells were pretreated with IGF1 or IGF2 neutralizing antibodies. Cells were then exposed to NNK for 5 and 15 min. Cell lysates were subjected to Western blot analysis.

a

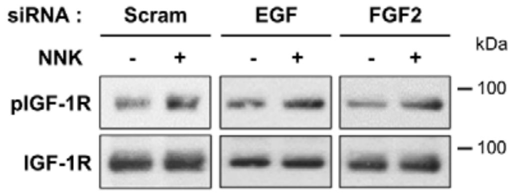

b

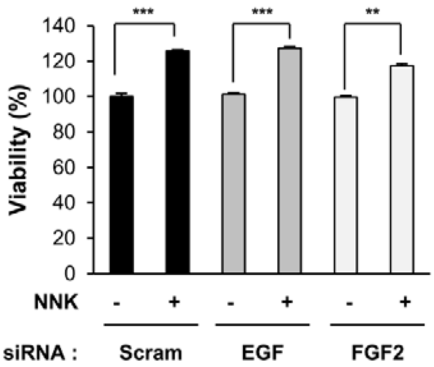

Supplementary Fig. 8. Effects of the ablation of FGF or EGF signaling on the NNK-induced increases in IGF-1R phosphorylation and cell viability. (**a**) BEAS-2B cells were transfected with scrambled siRNAs or siRNAs against EGF or FGF2 and then stimulated with NNK for 15 min. Cell lysates were determined by Western blot analysis. (**b**) BEAS-2B cells were transiently transfected with scrambled siRNAs or siRNAs against EGF or FGF2. NNK-mediated changes in cell viability were determined by the MTT assay.

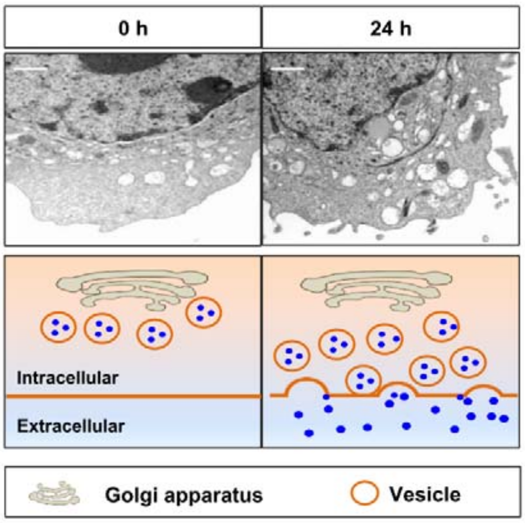

Supplementary Fig. 9. NNK can increase vesicle-like structure. Representative TEM images showing localization of vesicles after treatment with NNK for 24 h. Scale bar: 1 μm.

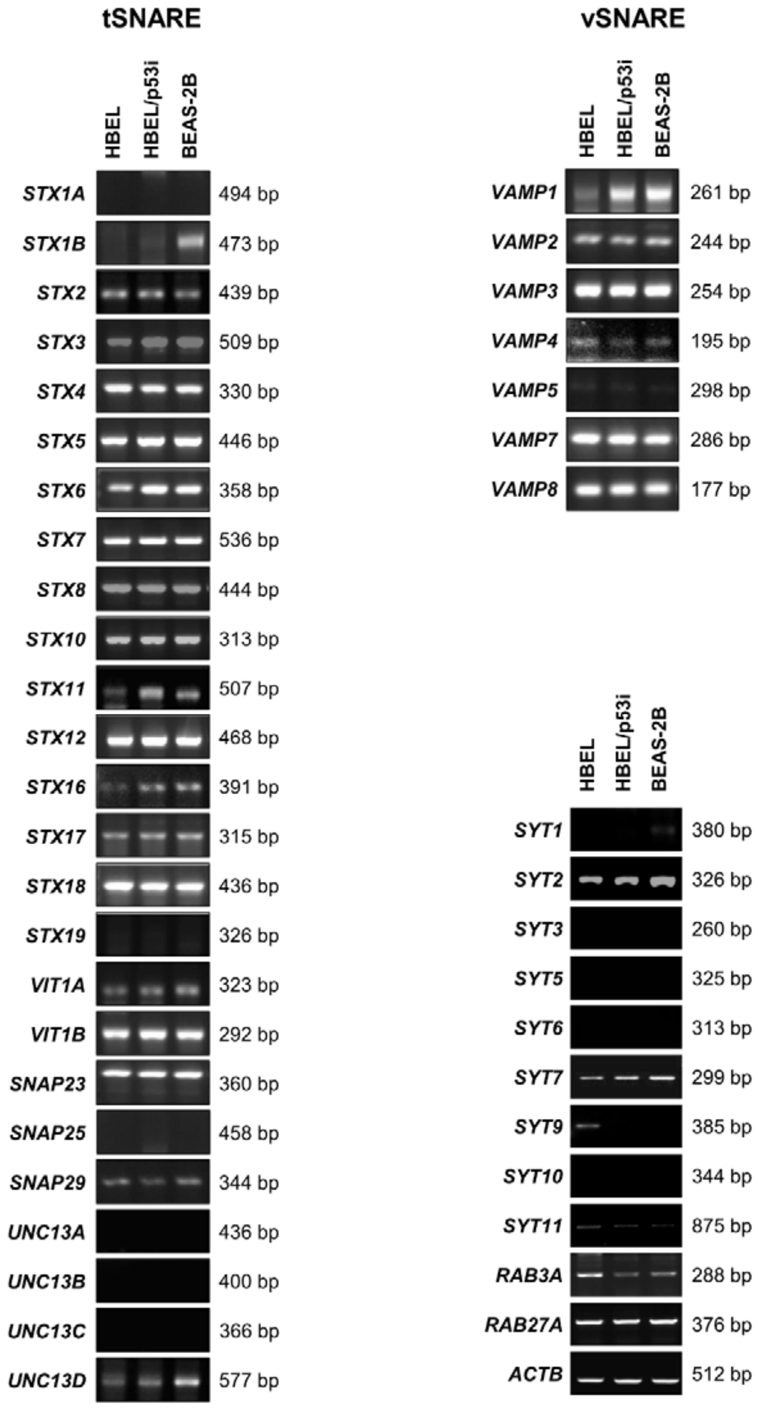

Supplementary Fig. 10. Basal expression of SNAREs, synaptotagmins, and Rabs in lung epithelial cells, determined by RT-PCR analysis.

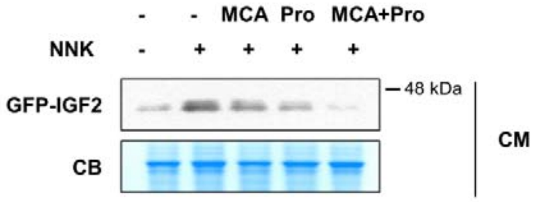

Supplementary Fig. 11. NNK induced IGF2 secretion *via* β-AR and nAChR. BEAS-2B cells were transfected with GFP-IGF2 expression vectors. After pretreatment with mecamylamine (10 μM), propranolol (10 μM), or both for 3 h, cells were further exposed to NNK (0.01 μM) for 15 min. CM from these cells were subjected to Western blot analysis.

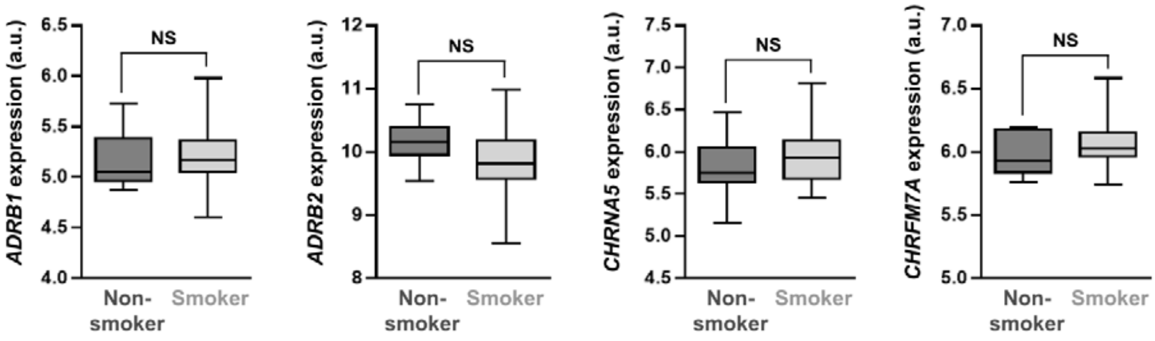

Supplementary Fig. 12. No significant difference in the expression of *ADRB1*, *ADRB2*, *CHRNA5*, and *CHRFM7A (CHRNA7)* expression between smokers and non-smokers. Representative results showing no significant difference in the *ADRB1*, *ADRB2*, *CHRNA5*, and *CHRFM7A (CHRNA7)* expressions between smokers and non-smokers. Analysis of a GEO dataset (GDS3257) to determine these gene expression in noninvolved normal lung tissues derived from patient with early stage (stage I-II) lung adenocarcinoma was performed by using GEO dataset analysis tools. NS: not significant compared with non-smokers.

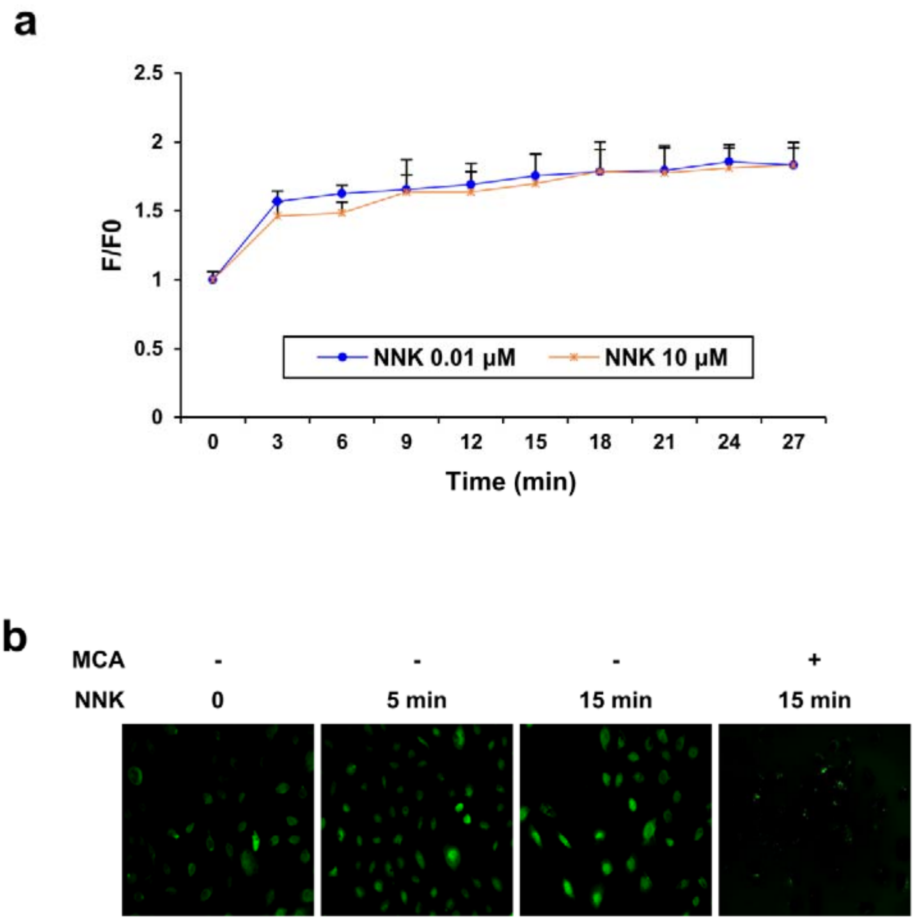

Supplementary Fig. 13. Increase in intracellular Ca^{2+} levels by treatment with NNK. (a) Time-lapse imaging of Fluo-4 AM fluorescence signals from HBEL/p53i cells exposed to NNK. (b) Fluo-4 AM-labeled HBEL/p53i cells, untreated or pretreated with mecamylamine (MCA), were exposed to NNK for 5 or 15 min. The green fluorescence signals were visualized using fluorescence microscopy.

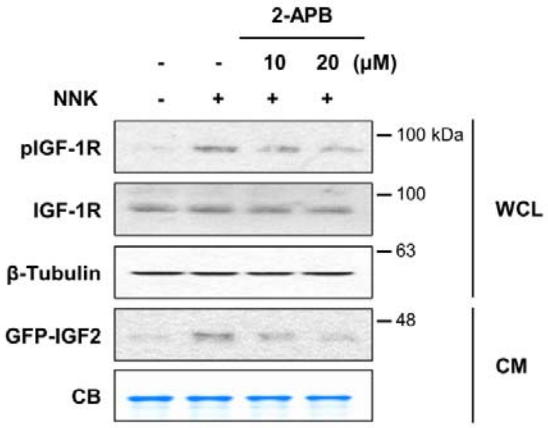

Supplementary Fig. 14. Blockade of IP_3 receptor partially inhibits NNK-induced IGF2 secretion and IGF-1R phosphorylation. BEAS-2B cells were pretreated with 2-APB for 3 h, and then exposed to NNK for 15 min. IGF-1R phosphorylation in WCL and IGF2 secretion in the CM were determined by Western blot analysis.

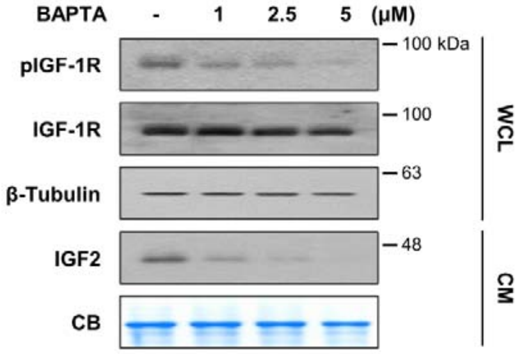

Supplementary Fig. 15. Basal intracellular Ca^{2+} level can induce IGF2 secretion and IGF-1R phosphorylation. BEAS-2B cells were treated with BAPTA-AM for 3 h. IGF-1R phosphorylation in WCL or IGF2 secretion in the CM were analyzed by Western blot analysis.

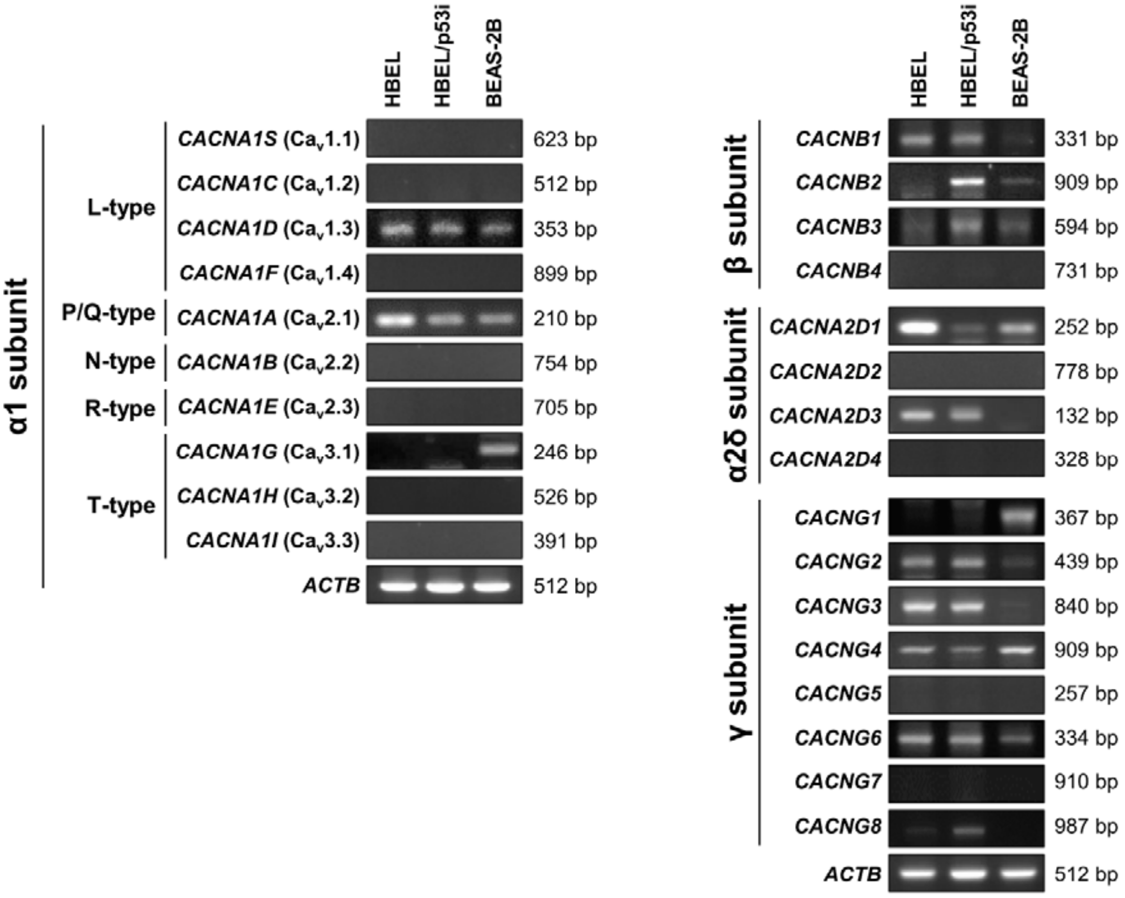

Supplementary Fig. 16. Basal expression of voltage-dependent calcium channel subunits in lung epithelial cells. The mRNA expression of voltage-dependent calcium channel subunits in lung epithelial cells was screened by RT-PCR analysis.

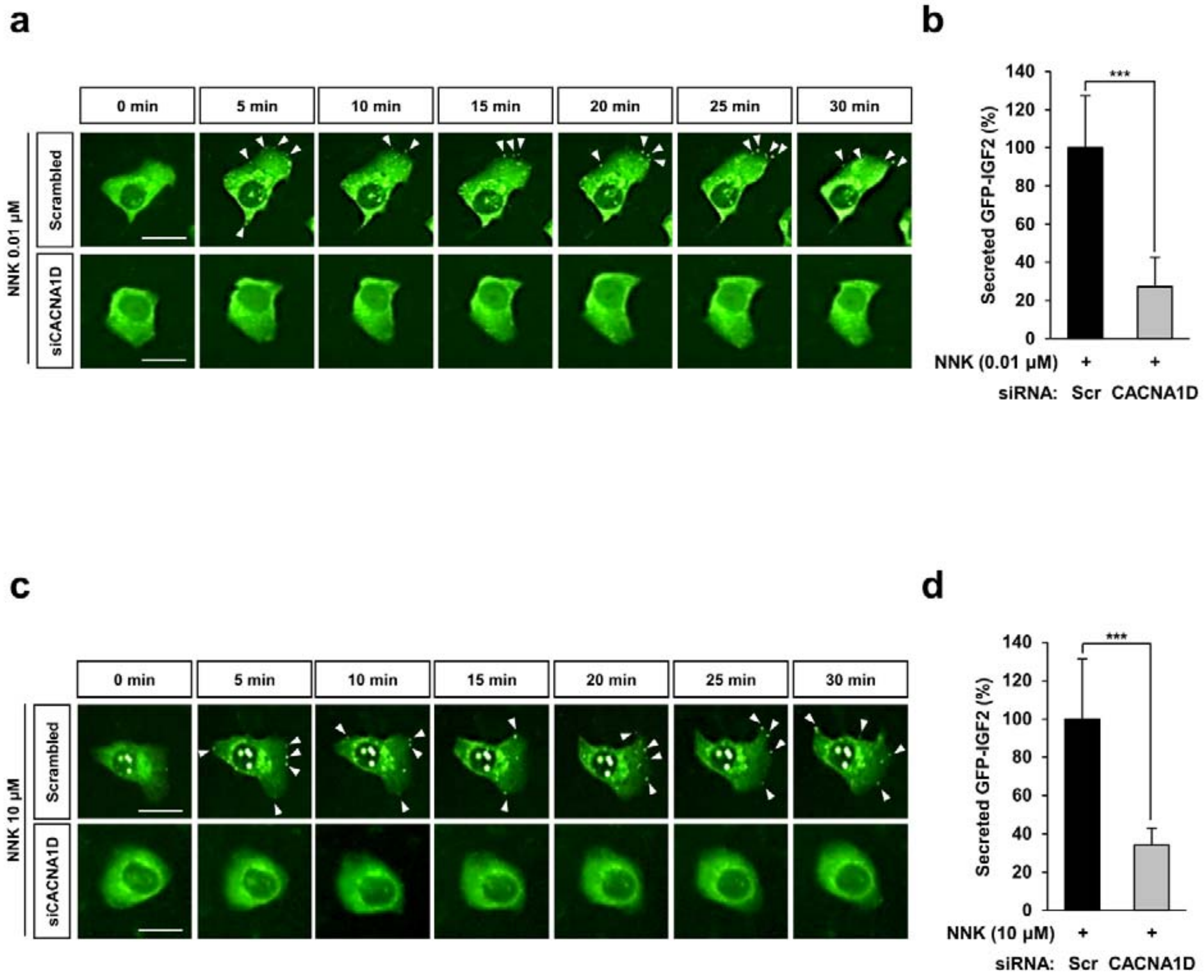

Supplementary Fig. 17. siRNA-mediated depletion of L-type calcium channel (CACNA1D) inhibits NNK-induced IGF2 secretion. (**a and c**) Time-lapse imaging analysis for GFP-IGF2 secretion from BEAS-2B cells. BEAS-2B cells were co-transfected with GFP-IGF2 and the indicated siRNA, and then stimulated with NNK (0.01 or 10 μM). (**b and d**) Quantification of secreted GFP-IGF2 at 30 min after NNK stimulation. Secreted GFP-IGF2 out of 25 BEAS-2B cells treated with NNK (0.01 or 10 μM) was quantified using Harmony high-content imaging and analysis software. Data are presented as the mean ± SD. *** $P < 0.001$, determined by Student's *t*-test. Scale bar: 20 μm.

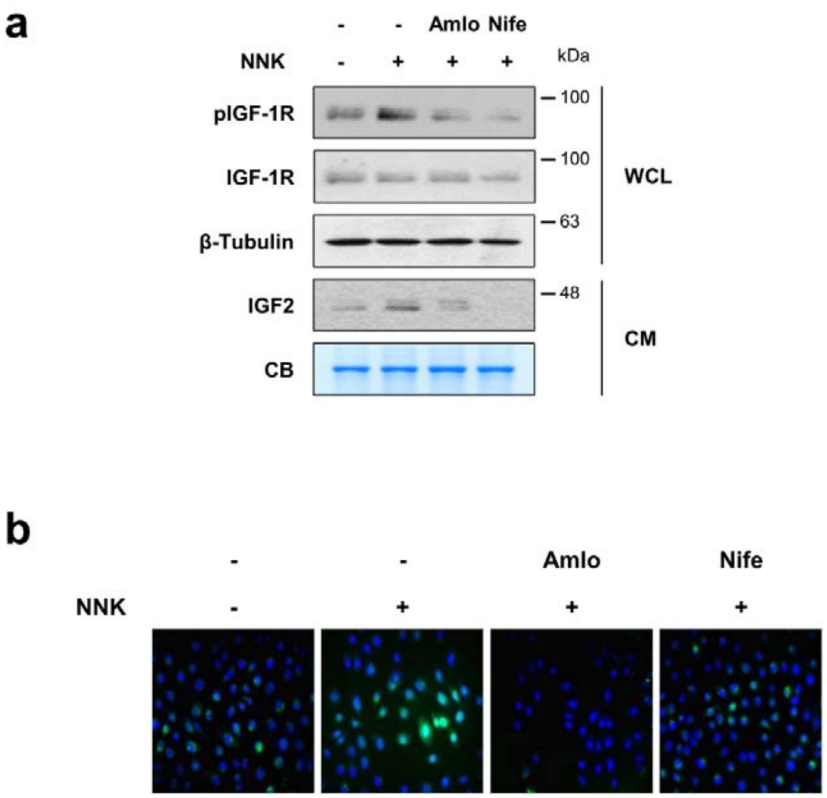

Supplementary Fig. 18. NNK-induced IGF2 secretion, IGF-1R phosphorylation and increase in intracellular Ca^{2+} level are suppressed by calcium channel blockers. (**a**) BEAS-2B cells were pretreated with amlodipine (10 μM) and nifedipine (10 μM) for 3 h, and then stimulated with NNK (0.01 μM) for 15 min. Whole cell lysates (WCL) and conditioned media (CM) from these cells were determined by Western blot analysis. (**b**) HBEL/p53i cells were pretreated with amlopdipine (10 μM) and nifedipine (10 μM) for 3 h, stained with Fluo-4 AM, and then exposed to NNK (10 μM) for 15 min. The green fluorescence signals were visualized using fluorescence microscopy. Amlo: amlodipine; Nife: nifedipine.

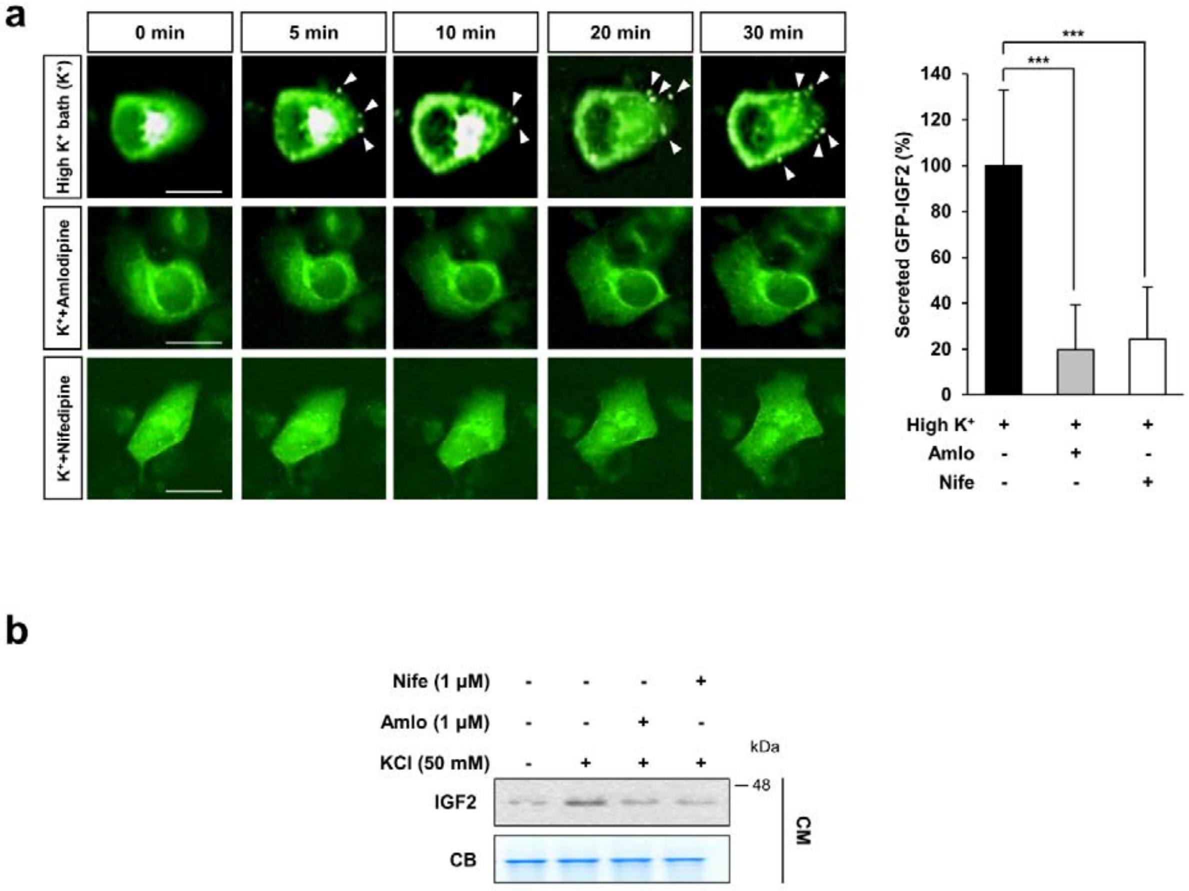

Supplementary Fig. 19. High [K^+]-mediated plasma membrane depolarization induces IGF2 secretion *via* L-type VDCC. (**a**) (left) Time-lapse imaging analysis for High K^+ bath-induced GFP-IGF2 secretion from BEAS-2B cells. BEAS-2B cells were transfected with GFP-IGF2 expression vectors, and then incubated with a high K^+ bath after pretreatment with amlodipine (1 μM) and nifedipine (1 μM) for 3 h. (right) Quantification of secreted GFP-IGF2 from 30 cells. Secreted GFP-IGF2 out of 30 BEAS-2B cells exposed to a High K^+ solution in the absence or presence of indicated inhibitors at 30 min after NNK treatment was quantified using Harmony high-content imaging and analysis software. Data are presented as the mean ± SD. *** $P < 0.001$, determined by Student's *t*-test. Scale bar: 20 μm. (**b**) BEAS-2B cells were transfected with GFP-IGF2 expression vectors. Cells were pretreated with amlodipine (1 μM) and nifedipine (1 μM) for 3 h, and were treated with KCl (50 mM) for 15 min. IGF2 secretion in the CM was subjected to Western blot analysis.

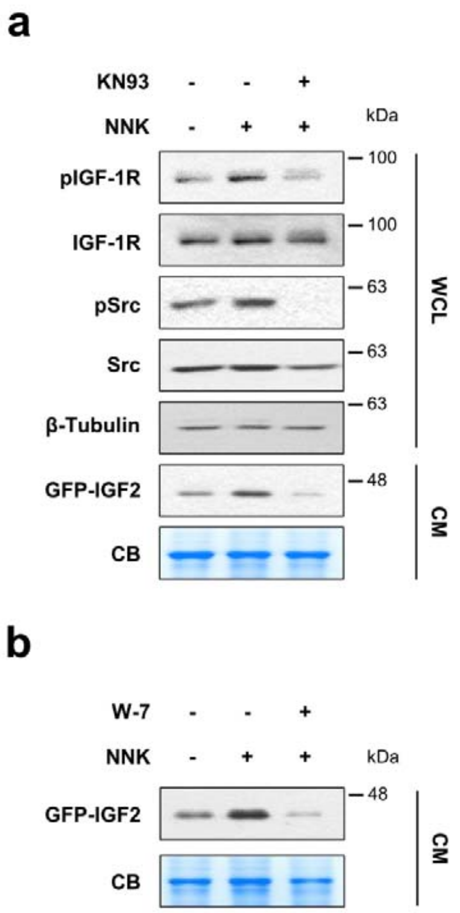

Supplementary Fig. 20. Calmodulin-mediated Src activation contributes to the NNK-induced IGF2 secretion an IGF-1R phosphorylation. BEAS-2B/GFP-IGF2 cells were pretreated with KN-93 (**a**) or W-7 (**b**) for 3 h and then stimulated to NNK for 15 min. The phosphorylation of IGF-1R and Src in WCL or IGF2 secretion in the CM were determined by Western blot analysis.

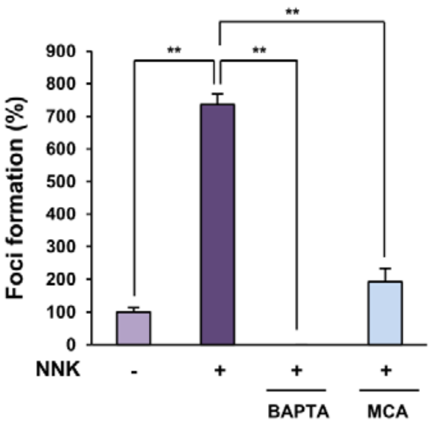

Supplementary Fig. 21. Blockade of NNK-induced foci formation by treatment with BAPTA-AM or mecamylamine. Quantification of foci formation in HBEL/p53i cells treated with NNK, alone or in combination with BAPTA-AM (BAPTA) or mecamylamine (MCA). Data are presented as the mean ± SD. **: $P < 0.01$, determined by Student's *t*-test.

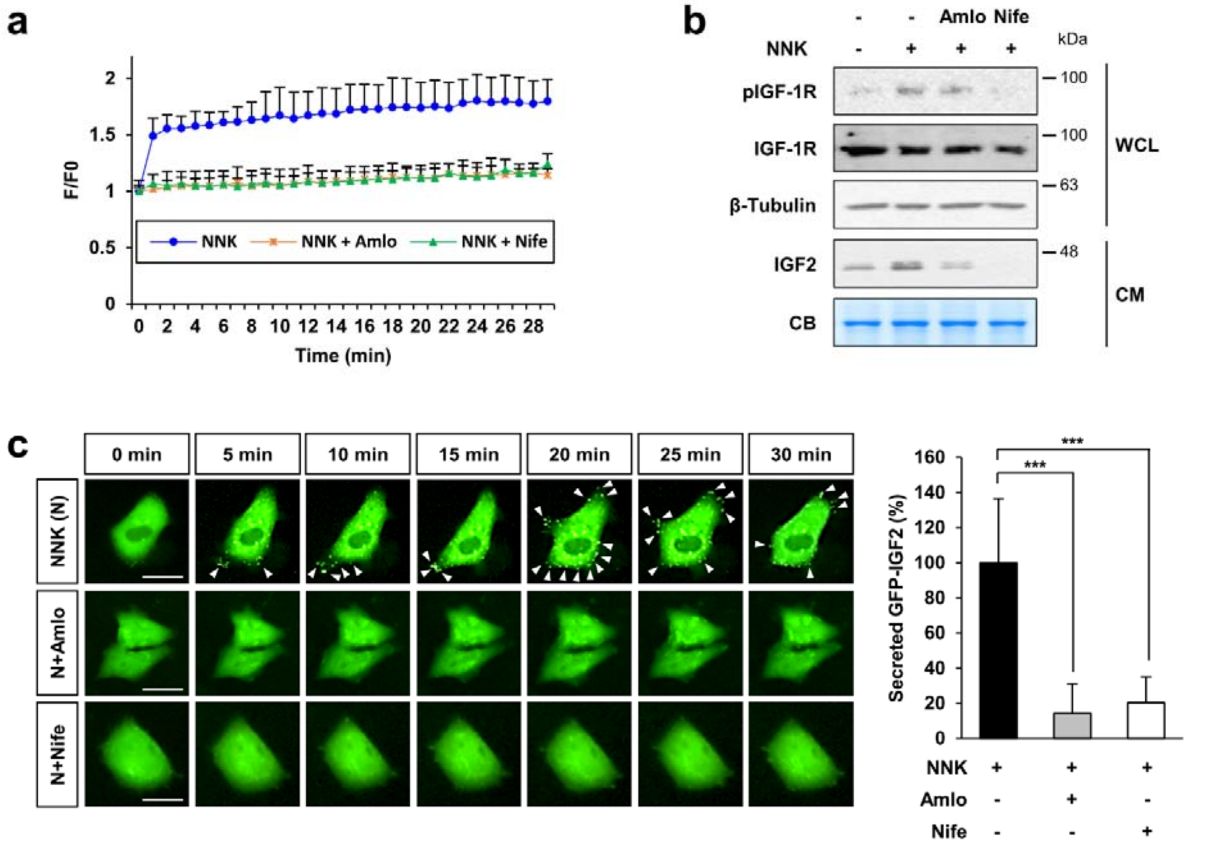

Supplementary Fig. 22. Low concentration of calcium channel blockers consistently inhibit NNK-induced intracellular Ca^{2+} level, IGF2 secretion, and IGF-1R phosphorylation. (**a**) Time-lapse imaging of Fluo-4 AM fluorescence signals from HBEL/p53i cells. Cells were pretreated with amlodipine (Amlo; 1 μM) or nifedipine (Nife; 1 μM) for 3 h, and were exposed to NNK (0.01 μM) for 30 min. The changes in fluorescence intensity were plotted to show the change in intracellular Ca^{2+} levels. Data were normalized to the average fluorescence intensity measured before NNK treatment in the HBEL/p53i cells. (n = 4, mean ± SD) (**b**) BEAS-2B cells transfected with GFP-IGF2 expression vectors were pretreated with amlodpine (1 μM) and nifedipine (1 μM) and then stimulated with NNK (0.01 μM) for 15 min. WCL and CM from these cells were determined by Western blot analysis. (**c**) *Left*. Time-lapse images of GFP-IGF2 secretion from BEAS-2B cells. BEAS-2B cells were transfected with GFP-IGF2 expression vectors. Cells were pretreated with amlodpine (1 μM) and nifedipine (1 μM) and then exposed to NNK (0.01 μM). *Right*. Quantification of secreted GFP-IGF2. Secreted GFP-IGF2 out of 25 BEAS-2B cells treated with NNK (0.01 μM) with or without indicated inhibitors at 30 min after NNK stimulation was quantified using Harmony high content imaging and analysis software. Data are presented as the mean ± SD. *** P < 0.001, determined by Student's t-test. Scale bar: 20 μm.

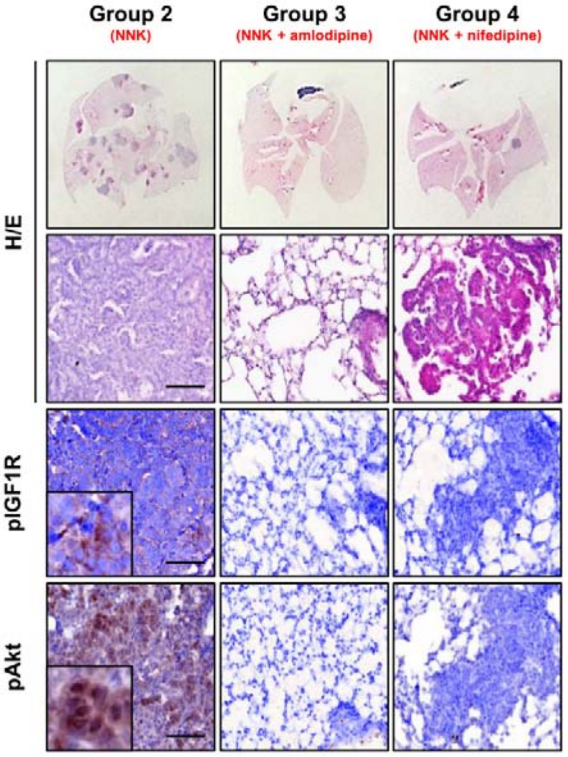

Supplementary Fig. 23. Regulation of the phosphorylation of IGF-1R and Akt in groups 2 (NNK), 3 (NNK + amlodipine), and 4 (NNK + nifedipine), determined by IHC analysis. Scale bar: 5 μm.

Fig. 3b

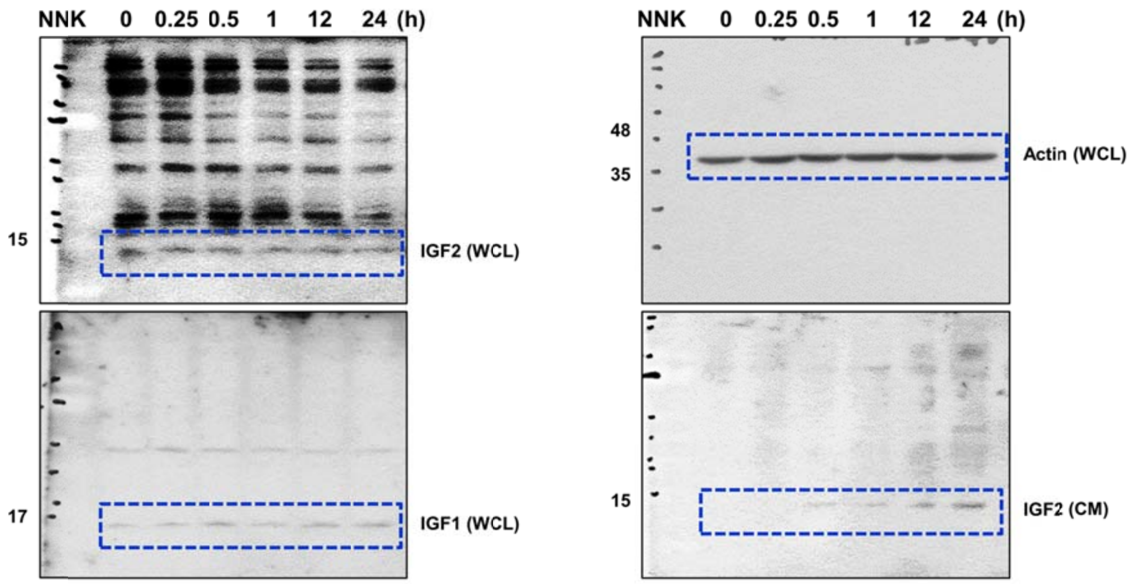

Fig. 3f

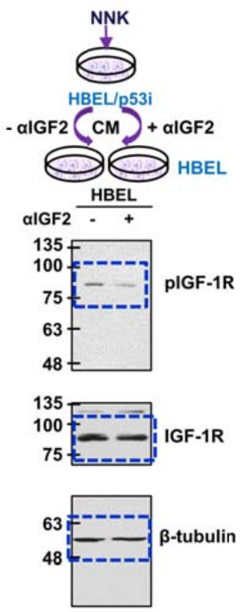

Fig. 4c

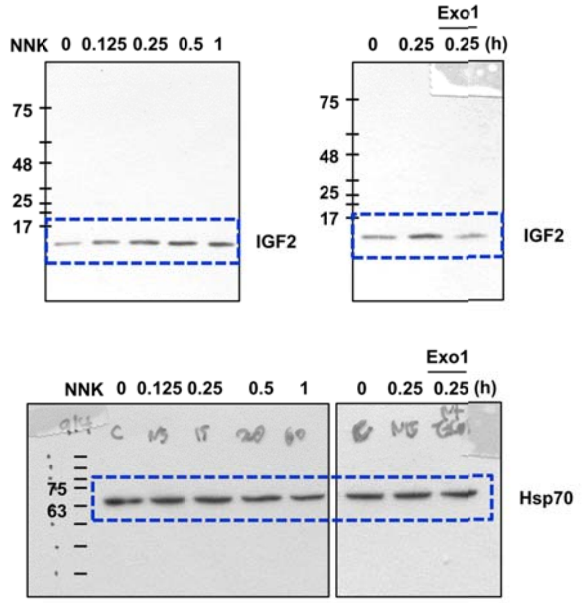

Supplementary Fig. 24. Uncropped, full Western blot images of important blots in indicates figures.

Fig. 4d

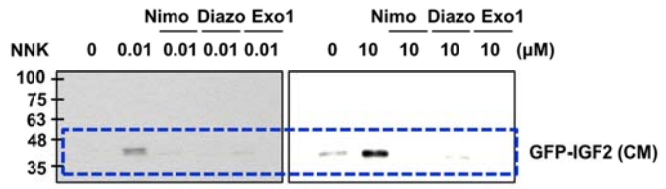

Fig. 4f

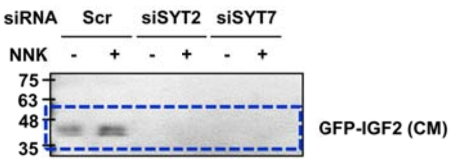

Fig. 4h

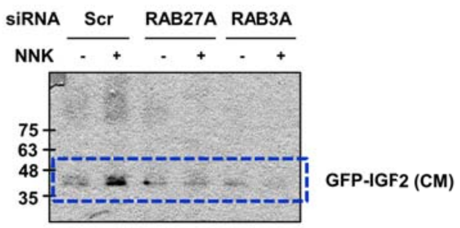

Fig. 5a

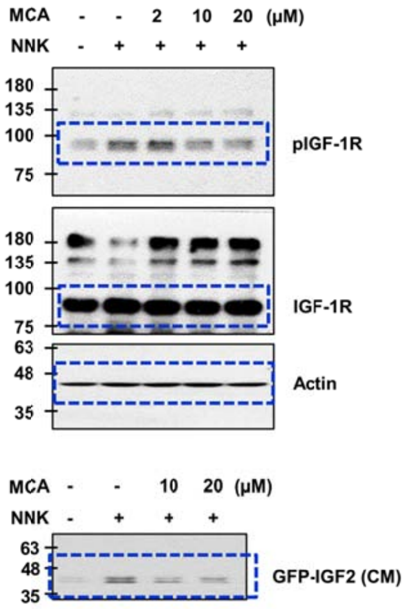

Fig. 5b

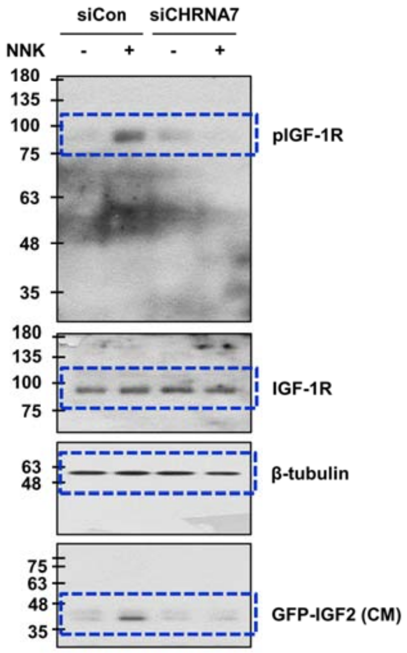

Supplementary Fig. 24. Continued.

Fig. 5c

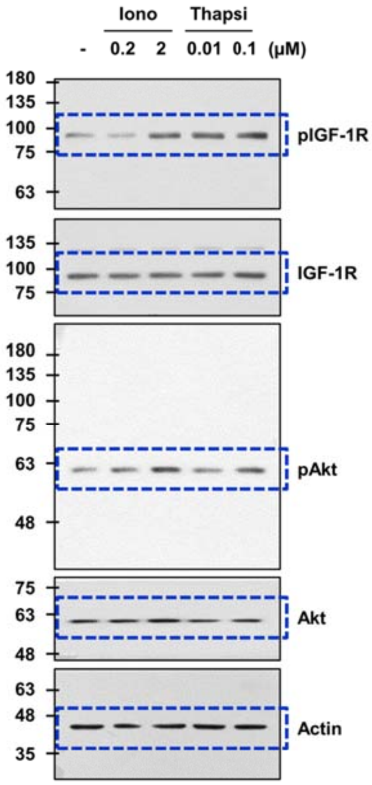

Fig. 5d

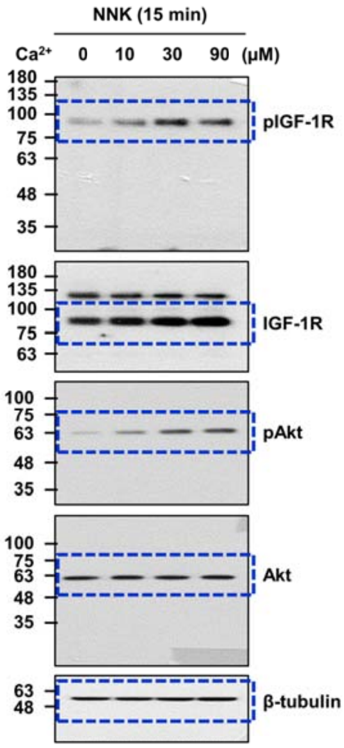

Fig. 5e

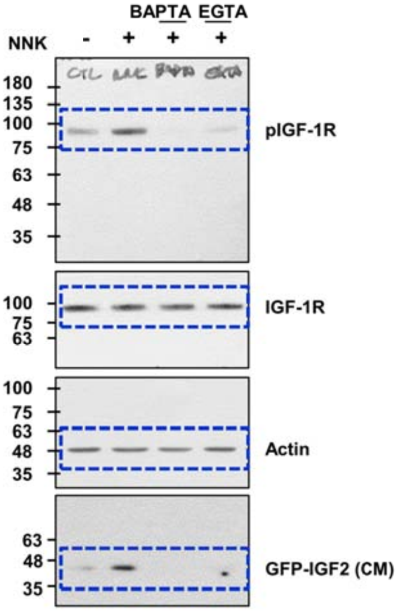

Fig. 5f

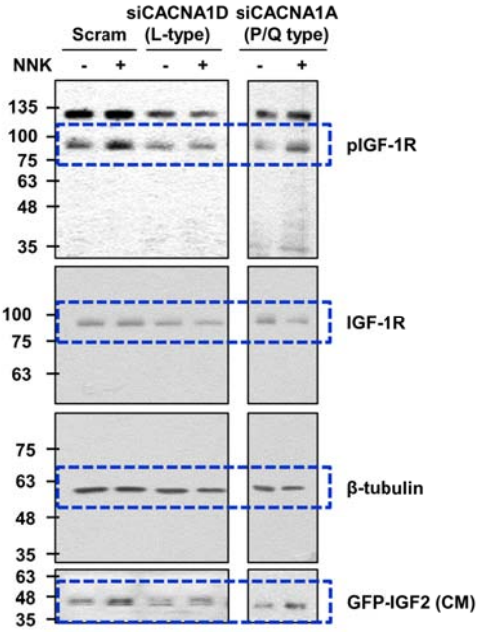

Supplementary Fig. 24. Continued.

Fig. 5g

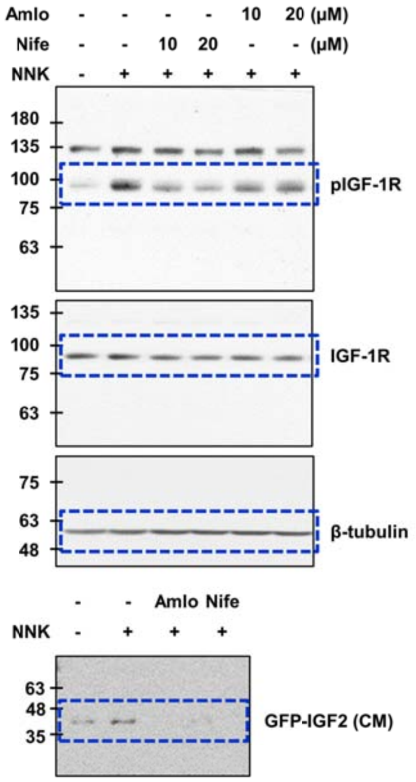

Supplementary Fig. 2

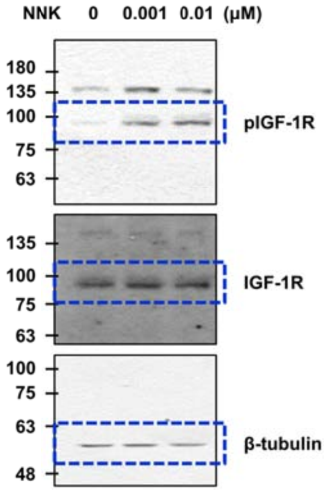

Supplementary Fig. 6b

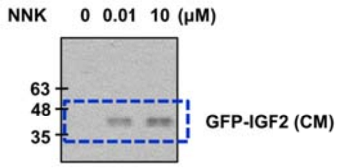

Supplementary Fig. 8a

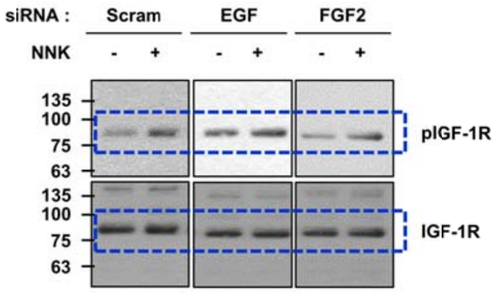

Supplementary Fig. 24. Continued.

Supplementary Fig. 11

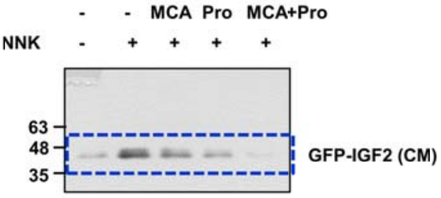

Supplementary Fig. 14

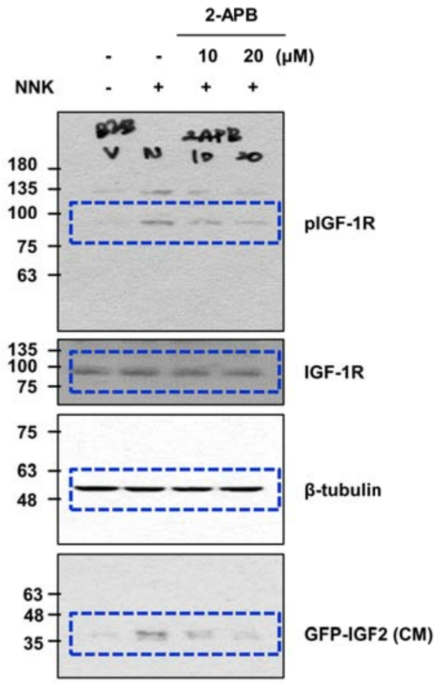

Supplementary Fig. 15

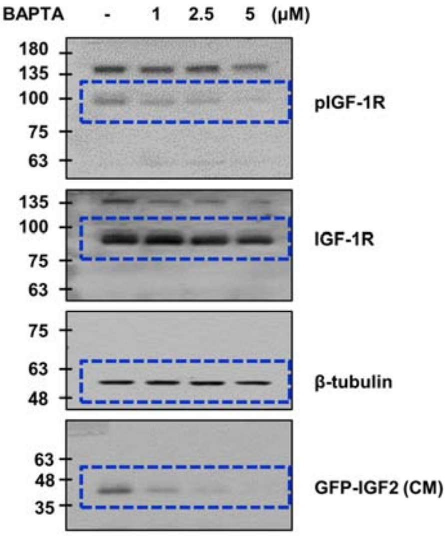

Supplementary Fig. 24. Continued.

Supplementary Fig. 18a

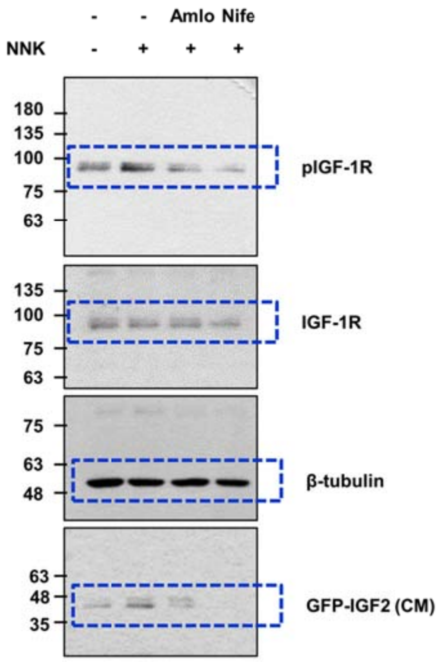

Supplementary Fig. 19b

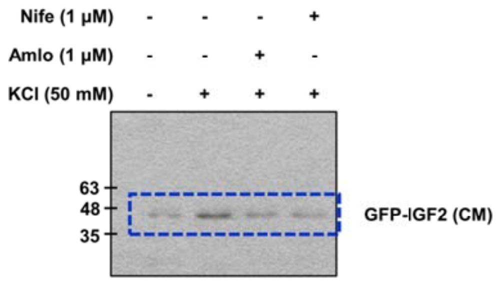

Supplementary Fig. 24. Continued.

Supplementary Fig. 20

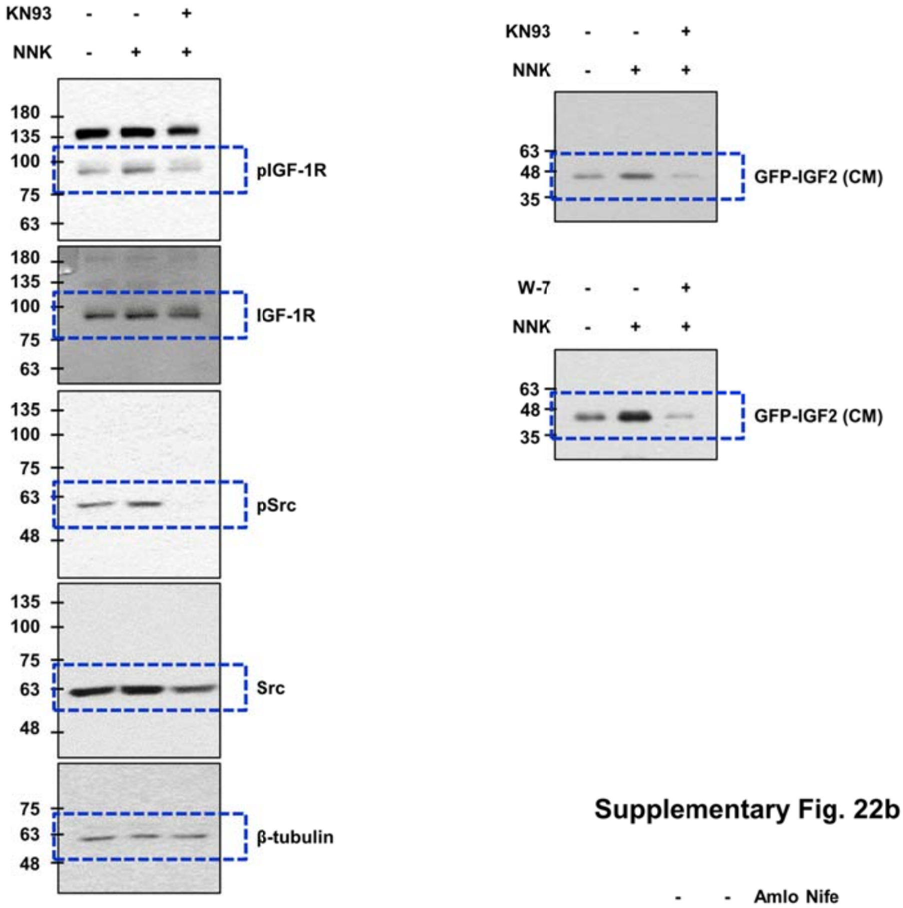

Supplementary Fig. 22b

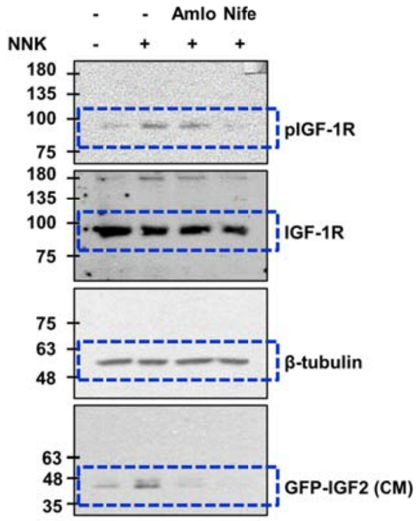

Supplementary Fig. 24. Continued.

Supplementary Table 1. Patients' demographic characteristics.

Type and histology of samples	No.	Gender[1]		Smoking history[2]		Smoking status[3]			Stage			
		F	M	Yes	No	Never	Former	Current	I	II	III	IV
**Total NSCLC**	**354**	**189**	**160**	**288**	**63**	**63**	**174**	**114**	**226**	**65**	**50**	**12**
Adenocarcinoma	235	143	92	176	58	58	105	71	160	28	36	10
Squamous cell carcinoma	119	46	73	112	5	5	69	43	66	37	14	2
**Total epithelial foci**	**367**	**123**	**205**	**206**	**41**	**41**	**99**	**104**	-	-	-	-
Normal	99	46	53	52	15	15	23	30	-	-	-	-
Hyperplasia	163	49	114	97	17	17	46	51	-	-	-	-
Squamous metaplasia	20	10	10	13	0	0	7	6	-	-	-	-
Low-grade dysplasia	29	4	24	18	2	2	9	5	-	-	-	-
High-grade dysplasia	56	14	42	26	7	7	14	12	-	-	-	-

[1]Gender information was not available in 5 NSCLCs and 39 epithelial specimens.

[2]Smoking history was not available in 3 NSCLCs and 120 epithelial specimens.

[3]Smoking status was not available in 11 NSCLCs and 123 epithelial specimens.

Supplementary Table 2. Total number of patients and the study population*.

	Total number of patients		Study population	
	Un-weighted	Weighted (95% CI)	Un-weighted	Weighted (95% CI)
NPS 2010	1.36	45.45 (45.40 – 45.51)	0.22	7.44 (7.41 – 7.46)
NPS 2011	1.37	45.51 (45.46 – 45.57)	0.23	7.62 (7.59 – 7.64)

*Study population includes all patients who were aged 20 years and older and had one or more records with hypertension and hypertension-related diagnoses. All figures are expressed in millions.

Supplementary Table 3. Demographic characteristics of the study population, NPS 2010-2011*.

	No. of population (frequency; %)	95% CI
**Overall population visits**	**15,054,548 (100)**	
**Gender**		
Male	7,060,496 (46.90)	7,060,432 – 7,060,553
Female	7,994,052 (53.10)	7,993,995 – 7,994,116
**Age group**		
20-34	246,679 (1.64)	246,667 – 246,699
35-49	2,152,615 (14.30)	2,152,559 – 2,152,680
50-64	5,886,502 (39.10)	5,886,449 – 5,886,554
65-79	5,460,185 (36.27)	5,450,785 – 5,469,588
80 and older	1,308,566 (8.69)	1,299,147 – 1,317,981
**Public Insurance Scheme**		
Health insurance	13,991,878 (92.94)	13,980-707 – 14,003,048
Medicaid	1,019,839 (6.77)	1,008,896 – 1,030,785
Veteran healthcare	42,831 (0.29)	40,497 – 45,164
**Calcium channel blocker**		
Dihydropyridines	8,484,325 (56.36)	8,462,613 – 8,506,030
Non-dihydropyridines	486,839 (3.23)	479,066 – 494,602
Lung cancer	89,631 (0.60)	86,248 – 93,007

*All figures are based on weighted analysis.

Supplementary Table 4. Primer sequences used for RT-PCR analysis.

Gene	Forward (5'-3')	Reverse (5'-3')
	**SNAREs** [1]	
STX1A	GGAGCTGGAAGAACTCATGT	CCACATTGTACTCGATCCTG
STX1B	GAGGATCTCACTGCAGACAT	ATGCGGTCAATCATCTCTCC
STX2	TTCTTTCTGCACCAAACCCG	GTCTCCAGCTTCATGATGTC
STX3	CACTCTGTCCTTTCTCGGAA	TTCAGCCCAACGGAAAGTCC
STX4	ATCCTGAAGGACACGCAGGT	TTATCCAACCACTGTGACGC
STX5	AACTGGCTTCTATGTCCAATG	GGTGACAGACTGGAAGTACT
STX6	AGTACTCGGCAAGTTGTCAG	ACTGAGTGCTCTCCAATTCG
STX7	GGAACACCTCAAGATTCACC	TGAACGTGCACCTCTGCATT
STX8	CCGTGACAATCAGAGCTTTG	GGTTGGCAAGGTCGTCAATT
STX10	TGAAGGACCATATGGTCAGC	TGGGTGTGGTCCATCTCTTG
STX11	CTTCTCGGTTCGCACTCTC	CAGTTGTCGCGCTGCTTCAT
STX12	CAGCTCGCCAAGGAAACAAA	CTCTGAGCTTTCCACATTGG
STX16	ATGACAGCAGCGAAGAGGAA	ATCTGGCGAATCTCTCGTTC
STX17	GAAGCATCAGCAGCAACAGC	GTCAATCTTCTCCTGCTGAG
STX18	CAAGGAGATACATTCCCAGC	GGAAATCTCAACCACTCTC
STX19	CTCTCAAAGGCAGGACTCCA	CTTCTCATTGAAGCCACCAG
VIT1A	ATCGCCTACAGTGACGAAGT	GCCAGAGACAGGTTACATTG
VIT1B	ATATATGCTGTAGAGAATGAGC	TGGAAAGCAGCAGCTTGTTG
SNAP23	CAAGACCATCACTATGCTGG	ACTTGAGTCAGGTTCTCTTC
SNAP25	TGCTGCAACTGGTTGAAGAG	CTTCTCCATGATCCTGTCGA
SNAP29	GAAGATGGTGGACAAGATGG	CTAGGTTGCTGTCGATCTTC
VAMP1	CTCCTCCTAACATGACCAGT	CTACCACGATGATGGCACAG
VAMP2	CCAAACCTCACCAGTAACAG	TGGCGCAAATCACTCCCAAG
VAMP3	GTCTACAGGTCCAACTGCTG	GAACAGTAATCCCGATTGCC
VAMP4	CCTTCGAAGTTGTTTGGATC	GGACCAAGATTTGGACCTAG
VAMP5	AGGAATAGAGTTGGAGCGGT	ACTACTGCTGTCACTGCTCT
VAMP7	CGGTTCAAGAGCACAGACAG	CTTCATACACATGGCTCGAG
VAMP8	GAAGGTGGAGGAAATGATCG	CTTCTGCGATGTCGTCTTGA
UNC13A	AATTGCCAAGCCACTCAGAC	CCAGAAGTCTGCCACTTGAG

UNC13B	CTGATCTTCACTGCTGCCAA	TCTGCCACTTGAGGTCATTG
UNC13C	GAGCACATGATTCGAGAGGA	GAACATTGCTGTGGTCTGCC
UNC13D	AGTCTGTCCTGCCTGAGGAT	AGGTCCTTCTTGTGCTTCTG
	**VDCC subunits** [2, 3, 4, 5]	
CACNA1C	GCCGCGCCGCAGTCAAGTCTA	GCTCCTCCGGGTCTGCATCTCATC
CACNA1S	CGCATCGTCAATGCCACCTGGTTTA	AGCACATTGTCGAAGTGGAAGTCGC
CACNA1D	GTGCCCTGCACACAGTAGTCGC	GGCGCGGGCAGGTCGGCTGTTGG
CACNA1F	AGGGACCCCTAAGCGAAGAAACCAG	ACCCCATGGCATCTTGCATCCAGTA
CACNA1A	TCCTCAAGCATTCGGTGGAC	TGCAGGCCCTCTCATTTTTC
CACNA1B	GGAACTGACTTCGACCTGCGAACAC	CCTCCTCTGCGTGGATCAGGTCAT
CACNA1E	TCTGCCATCTACTTCATTGTG	CTCCTTGCCTTCTGTCCT
CACNA1G	TGTCTCCGCACGGTCTGTAA	AAGCCGGTTCCAAGTGTCTC
CACNA1H	CCTGATCCCTACGAGAAGATCCCGC	CACGGCTGAAGTACTTGCTGTCCAC
CACNA1I	AGATGCCCTTCATCTGCTCCCTGTC	AAGATCTCCTCGTAGCAGTCGCCAG
CACNB1	ATGCACGAGTACCCAGGGGAG	CAGCGCAGTAGCGGGCCTTATT
CACNB2	TCGCTTGCCAAACGCTCGGT	ATGACGGCTGCGCTGCTTGT
CACNB3	GCAGCAGCTCGAAAGGGCCA	ATGCTGGAGCGGGCAGAGGA
CACNB4	TGAAGACTCGGAGGCTGGTTCAGC	TGGACCGGGTGTTCGAACGT
CACNA2D1	TGCTCATCGGCCCCTCGTCG	CCAGGCGCACCAGGGCTTTAG
CACNA2D2	AGCCTAGGCAGGCGCACACT	TCTGCACTAGCTCACACTGCTCCGG
CACNA2D3	GGACGAGAGGCTGCGTTTGCA	GGGCCGGCTAAGCACGTGAA
CACNA2D4	TGGCCTGGGCCTTTGTGCAG	GCCTCCTCGGCAGCTTCCAC
CACNG1	TGCTGGCCATGACAGCCGTG	AACATGGACGCGGGTCGCAG
CACNG2	TCTCTGGGCCTTAATTTTCCCC	TTTTCACAGACCCCCAAAGACA
CACNG3	CTCCCCTTCCCCTTTCCTTAAC	AGCTGGGATTTCCTTTCTGGAG
CACNG4	TTTGCACGAAGGTTGTGCTG	TTGCTCTCCTGGCGTTGATT
CACNG5	GATCAAGATGTCCCTGCACTCA	CAGAGACAAAGGCCAGTATCGT
CACNG6	TGCTCAGTAAAGGTGCAGAGTT	CTCGGTGGTTGCTTAGAGAAGT
CACNG7	ACTGGCTGTACATGGAAGAAGG	TGAAATAAGGGAGTCTGTGGGC
CACNG8	TGCTGAAGCATAGTCATGGTGT	CCTCTGCCTTCTCAGTGAACTT
	**Synaptotagmins**	
SYT1	CGCCCCTGTCACCACTGTTGC	CCTTGGGCTCTTCCTTTTCTTCTC
SYT2	GCTCCGGCCACCACCACTG	GTCTCCGCGTCGTCATCATCCTG

SYT3	ACCATCCACCATTTGCTGAACTG	GGTGGTGGGTGCTAGACTTGTGA
SYT5	GGATTGGCAGCCTTGGATCT	CCCCAAGCTTCTCCTCCTCC
SYT6	TCCCTACTATGTGATGGGCG	GGGTTCCCTCTTTGAAGGATTT
SYT7	ATCACCGTCAGCCTTAGCGTCACT	TCGGAGCCTGGGGAGAGCAT
SYT9	GGCAGGCTGACCATTACCAT	CCCATGGTCATCGTTTCTCCA
SYT10	GCTTTTGTGGACTGGCCTTG	TCTTCGGAAGGAACTGTGGC
SYT11	TCGGTGACCGTCTTTGTCTG	TCCATCTTCGGCAAGTGTCT
	**Others**	
RAB3A	TTCCGCTATGCTGACGACTC	GTTTCCTACCAGCAGCACCT
RAB27A	AGAGGCCAGAGAATCCACCT	CACACCGTTCCATTCGCTTC
CHRNA7	GTCCCTGCAAGGCGAGTT	TATGCCTGGAGGCAGGTACT
ACTB	ACTACCTCATGAAGATC	GATCCACATCTGCTGGAA

Supplementary Table 5. Primer sequences used for real-time PCR analysis.

Gene	Forward (5'-3')	Reverse (5'-3')
IGF1	ATGTATTGCGCACCCCTCAA	GGGCACGGACAGAGCG
IGF2	CCGTGCTTCCGGACAACTT	CTGCTTCCAGGTGTCATATTGC
IGF1R	TGAAAGTGACGTCCTGCATTTC	GGTACCGGTGCCAGGTTATG
INSR	GAGACGCAGAGATGCAGC	AGGAGCCCAATGGTCTGA
ACTB	GCGAGAAGATGACCCAGATC	GGATAGCACAGCCTGGATAG

Supplementary References

1. Pattu V*, et al.* SNARE protein expression and localization in human cytotoxic T lymphocytes. *European journal of immunology* **42**, 470-475 (2012).

2. Park JY*, et al.* Molecular identification of Ca2+ channels in human sperm. *Exp Mol Med* **35**, 285-292 (2003).

3. Sousa SR, Vetter I, Ragnarsson L, Lewis RJ. Expression and pharmacology of endogenous Cav channels in SH-SY5Y human neuroblastoma cells. *PloS one* **8**, e59293 (2013).

4. Sheppard BJ, Williams M, Plummer HK, Schuller HM. Activation of voltage-operated Ca2+-channels in human small cell lung carcinoma by the tobacco-specific nitrosamine 4-(methylnitrosamino)-1-(3-pyridyl)-1-butanone. *Int J Oncol* **16**, 513-518 (2000).

5. Yu Y, Richardson DR. Cellular iron depletion stimulates the JNK and p38 MAPK signaling transduction pathways, dissociation of ASK1-thioredoxin, and activation of ASK1. *The Journal of biological chemistry* **286**, 15413-15427 (2011).
